# Acylglycerol kinase inhibits macrophage anti-tumor activity via limiting mtDNA release and cGAS-STING-type I IFN response

**DOI:** 10.7150/thno.101298

**Published:** 2025-01-01

**Authors:** Qiuyang Du, Na Ning, Xiujuan Zhao, Feifan Liu, Si Zhang, Yuting Xia, Fei Li, Shijie Yuan, Xiaorong Xie, Mengdi Zhu, Zehan Huang, Zhaohui Tang, Jing Wang, Ran He, Xiang-Ping Yang

**Affiliations:** 1 Department of Immunology, School of Basic Medicine, Tongji Medical College, Huazhong University of Science and Technology, Wuhan, China.; 2 The Second Affiliated Hospital of Guangzhou Medical University, Guangdong Provincial Key Laboratory of Allergy & Clinical Immunology, Guangzhou 510260, China.; 3 Department of Pathology, Renmin Hospital of Wuhan University, Wuhan, 430060, China.; 4 Department of Pathology, Tangdu Hospital, Air Force Medical University, Xi'an, Shaanxi, China.; 5 Department of Otolaryngology-Head and Neck Surgery, Tongji Hospital, Tongji Medical College, Huazhong University of Science and Technology, Wuhan, China.; 6 Institute of Allergy and Clinical Immunology, Tongji Hospital, Tongji Medical College, Huazhong University of Science and Technology, Wuhan, China.; 7 Hubei Clinical Research Center for Nasal Inflammatory Diseases, Wuhan, China.; 8 Department of Cardiology, Union Hospital, Tongji Medical College, Huazhong University of Science and Technology; Wuhan, China.; 9 Department of Dermatology, Union Hospital, Tongji Medical College, Huazhong University of Science and Technology (HUST), Wuhan, China.; 10 Division of Trauma Surgery, Department of Surgery, Tongji Hospital, Tongji Medical College, Huazhong University of Science and Technology, Wuhan, China.

**Keywords:** TAMs, cGAS-STING-type I IFN signaling pathway, Acylglycerol kinase, Mitochondrial ROS, mitochondrial DNA

## Abstract

**Background**: Tumor associated macrophages (TAMs) are critical components in regulating the immune statuses of the tumor microenvironments. Although TAM has been intensively studied, it is unclear how mitochondrial proteins such as AGK regulate the TAMs' function.

**Methods**: We investigated the AGK function in TAMs using macrophage-specific *Agk* deficient mice with B16 and LLC syngeneic tumor models. Flow cytometry was used to evaluate the stemness and activation of CD8^+^ T cells. The enhanced release of mtDNA into the cytosol in the *Agk*-deficient BMDMs was measured by RT-PCR and immunofluorescence; the cGAS-STING-type I IFN pathway was evaluated by immunoblotting. Mitochondria functions were evaluated by electron microscope and seahorse equipment.

**Results**: We have noted an increased expression of AGK in TAMs of multiple tumor types, which was negatively correlates with the tumor tissue immune scores. In the B16 and LLC tumor models, macrophage *Agk*-deficient mice have reduced tumor growth and enhanced populations of CD8^+^ Tpex. AGK-deficient macrophages have increased mitochondrial damage and mtDNA release into the cytosol, which leads to enhanced cGAS-STING-type I IFN activation. Blockade of the type I IFN signaling pathway with anti-IFNAR reversed the phenotype in *Agk*-deficient mice.

**Conclusions**: Our findings define a critical role of AGK in maintaining the macrophage mitochondrial homeostasis that is associated with mtDNA release and following cGAS-STING activation and type I IFN pathway. Targeting AGK in TAMs may represent a novel strategy to enhance anti-tumoral activity.

## Introduction

Tumor associated macrophages (TAMs) are the most abundant immune cells infiltrated within tumors 1. In general, TAMs are considered to promote tumor progression and exhibit an M2 phenotype. This phenotype is characterized by an increased expression of Arg1 and a reduced expression of MHC II in mouse models, or increased expression of CD204, CD163, and CD206 in human subjects 2. These M2 TAMs are potent regulators of tumor-associated immune suppression in the tumor microenvironment (TME) 3. Various therapeutic strategies aimed at targeting TAMs, such as depleting TAMs, inhibiting monocyte recruitment, and inducing phenotypic reprogramming have been intensively studied 4. However, these approaches have been of limited clinical success, underscoring the intricate nature of TAM biology 5.

Recent single-cell analysis of numerous human tumors and mouse models has revealed a high degree of heterogeneity among TAMs. Some TAMs have been reported to inhibit tumor growth via phagocytosis of tumor cells, secretion of proinflammatory cytokines and antigen presentation of tumor antigens to effector T cells 6. A recent study showed that TAMs with downregulated Ly6C and enhanced MHC-II expression have anti-tumor activity referred as mature TAMs (mTAMs), while TAMs that expressing high Ly6C and low levels of MHC-II (immature TAMs, iTAMs) are pro-tumoral 7. It is felt that these functionally distinct TAM populations are closely regulated by molecules in the TME, including various cytokines, nucleic acids, damage-associated molecular patterns (DAMPs) and metabolites 8, 9. Vismodegib, a pharmacologic Hedgehog signaling inhibitor, induced a significant shift of TAMs to inflammatory phenotype through altering metabolic processes in mouse tumor models 10. Other exogenous small molecules, such as HDAC-II inhibitors, can reprogram TAMs into a pro-tumoral phenotype 11. Recently, it has been shown that TLR agonists and tumor exosome vesicles can also polarize the TAMs to anti-tumor phenotypes 12, 13. Nevertheless, it remains unclear whether TAMs can be intrinsically regulated by specific molecular pathways. Mitochondria are catabolic organelles and are the major source of cellular ATP and ROS, which have essential functions in both innate and adaptive immunity, and mitochondria-mediated metabolic changes are associated with TAMs polarization 14. Additionally, mitochondrial DNA (mtDNA) as an important DAMP released by damaged mitochondria, plays an important role in the recruitment, survival, polarization, and function of macrophages 8. Targeting mitochondria homeostasis represents a promising strategy to switch TAMs from pro-tumor toward anti-tumor function for cancer therapy. Acylglycerol kinase (AGK) is a mitochondrial acylglycerol kinase, which catalyzes the production of lysophosphatidic acid and phosphatidic acid from monoacylglycerol and diacylglycerol 15. Its mutations can cause Sengers syndrome characterized by congenital cataracts, hypertrophic cardiomyopathy, skeletal myopathy, and lactic acidosis due to the impaired mitochondrial function 16. Apart from its kinase activity, AGK contributes to the mitochondrial inner membrane 22 (TIM22) complex with other proteins, which is essential to maintain mitochondrial structure and function 17. In CD8^+^ T cells, membrane localized AGK phosphorylates and inactivates PTEN (Ser380, Thr382, and Thr383). This leads to activation of the PI3K/AKT signaling pathway, which is required for the full anti-tumor function of CD8^+^ T cells 18. We recently found AGK negatively regulates the sensitivity of diffuse large B cell lymphoma (DLBCL) to Venetoclax by phosphorylating PTEN, thus activating PI3K/AKT and in turn inhibiting FOXO1 and enhancing BCL-2 19. AGK has been reported to be upregulated in prostate cancer and breast cancer and participates in the tumor proliferation and progression through activating the PI3K/AKT/GSK3β signaling 20, 21. Given the significant role of AGK in tumor progression, defining the roles of AGK in TAMs may provide insights on how to further enhance the efficacy of immunotherapy.

In this study, we investigated the function of AGK in macrophages using mice with specific deletion of AGK in macrophages in B16-F10 melanoma and LLC tumor models. We found that AGK deficiency in macrophages stimulated a transformation towards an anti-tumorigenic phenotype, leading to enhanced T cell infiltration, increased stemness, and improved anti-tumor activity. Mechanistically, AGK deficiency led to the activation of the cGAS-STING signaling pathway and subsequent type I IFN response by inducing mitochondrial damage and the accumulation of cytoplasmic mitochondrial DNA (mtDNA). Inhibition of STING abolished the enhanced type I IFN response in AGK-deficient macrophages and blockade of type I IFNs eradicated the enhanced anti-tumoral activity in *Agk^cKO^* mice. These findings underscore the potential of targeting AGK as a novel strategy to augment the cGAS-STING-type I IFN axis within macrophages, thereby bolstering their anti-tumor activity.

## Results

### AGK expression is negatively associated with immune scores and upregulated in TAMs in multiple types of tumors

**Figure 1:**
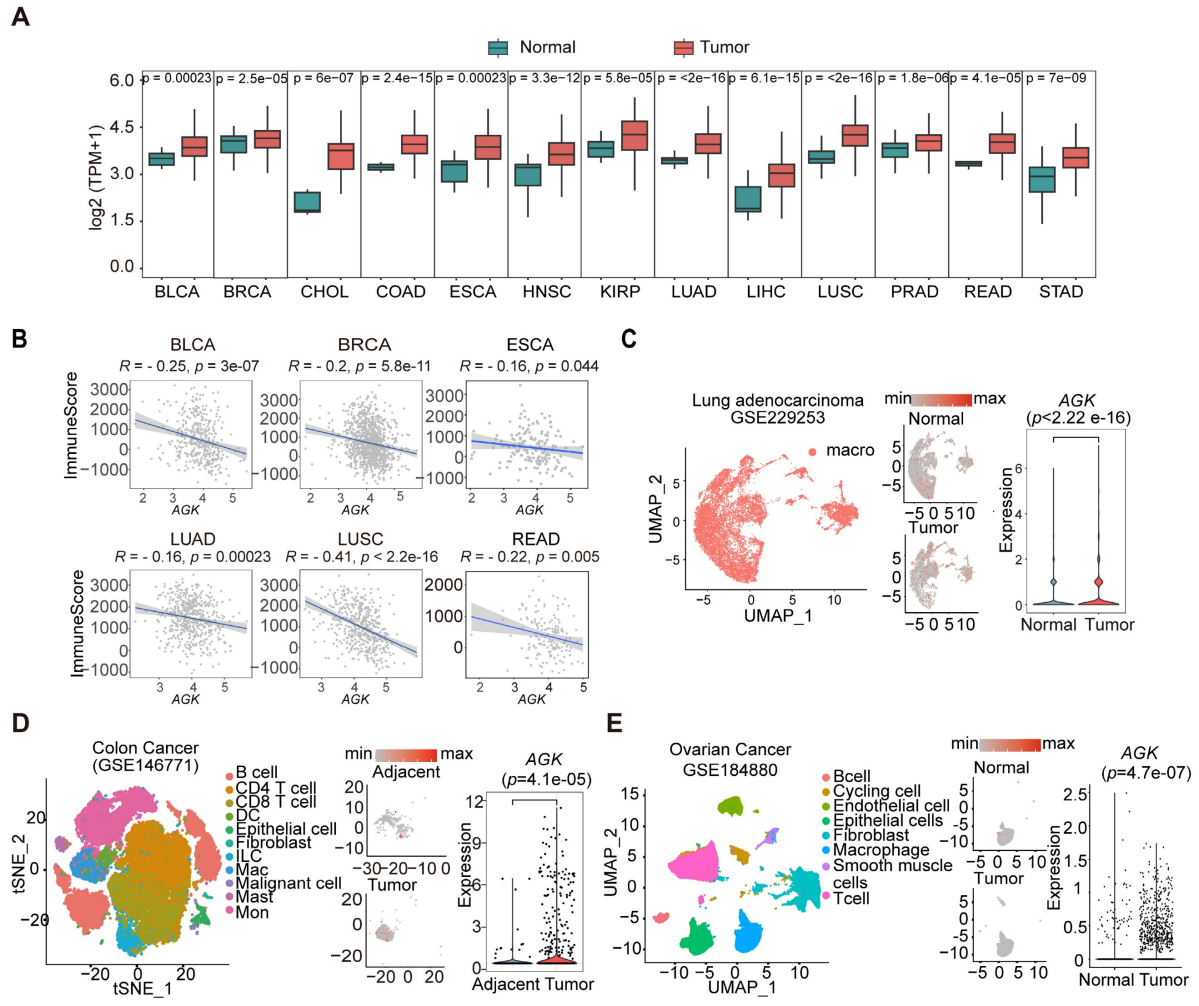
** AGK expression is negatively associated with immune scores and upregulated in TAMs in multiple types of tumors.** (**A**) Boxplots showing the AGK expression in multiple types of cancers and corresponding normal tissues from RNA-seq results of the TCGA database. Red and Green boxes indicate the tumor group and normal group, respectively. The horizontal line indicates the median value; the box represents the first and third quartiles. Data were analyzed by two-sided unpaired wilcoxon test. (**B**) Scatter plots showing an inverse Spearman's correlation between AGK expression and immune scores calculated by the ESTIMATE algorithm from the TCGA database. Data are shown as a fitted line with 95% confidence level and were analyzed by linear model. (**C-E**) AGK expression in tumor-infiltrating macrophages and corresponding normal tissues from single-cell RNA-seq datasets of LUAD (**C**), COAD (**D**) and OV (**E**). We re-clustered the identified macrophages and analyzed AGK expression in those cells in normal and tumor tissues to enhance the visual appeal while preserving the gene expression comparisons (**D**-**E**). P values were calculated by two-sided unpaired wilcoxon test. Abbreviation for TCGA cancer types: BLCA, Bladder urothelial carcinoma; BRCA, Breast invasive carcinoma; CHOL, Cholangiocarcinoma; COAD, Colon adenocarcinoma; ESCA, Esophageal carcinoma; HNSC, Head and Neck squamous cell carcinoma; KIRP, Kidney renal papillary cell carcinoma. LUAD, Lung adenocarcinoma; LIHC, Liver hepatocellular carcinoma; LUSC, Lung squamous cell carcinoma; PARD, Prostate adenocarcinoma; READ, Rectum adenocarcinoma; STAD, Stomach adenocarcinoma.

We screened the expression of AGK in tumors and adjacent normal tissues from RNA sequencing (RNA-seq) results of The Cancer Genome Atlas (TCGA) database and the Genotype Tissue Expression (GTEx) database. In keeping with previous reports, we found the expression of AGK mRNA was significantly upregulated in 13 of 24 types of tumor tissues, including breast cancer (BRCA) and adenocarcinomas of the colon (COAD), rectum (READ), stomach (STAD), and prostate (PRAD) (Figure 1A) 20, 21. Using the estimation of stromal and immune cells in malignant tumor tissues using expression data (ESTIMATE) algorithm, we found a negative correlation between the expressions of AGK mRNA and immune scores in BRCA, Bladder Urothelial cancer (BLCA), Esophageal cancer (ESCA), Lung squamous cell cancer (LUSC), Lung adenocarcinoma (LUAD) and Rectum adenocarcinoma (READ) (Figure 1B). Next, we compared the expression in TAMs and macrophages in the adjacent tissues with public single-cell RNA-seq (scRNA-seq) data and found that TAMs expressed higher AGK than macrophages in adjacent or normal tissues in individuals with LUAD (gse229253), COAD (gse146771), and OV (gse184880) (Figure 1C-E), suggesting a possible regulatory role of AGK in TAM function.

### Deletion of AGK in macrophages leads to enhanced anti-tumor activity

**Figure 2:**
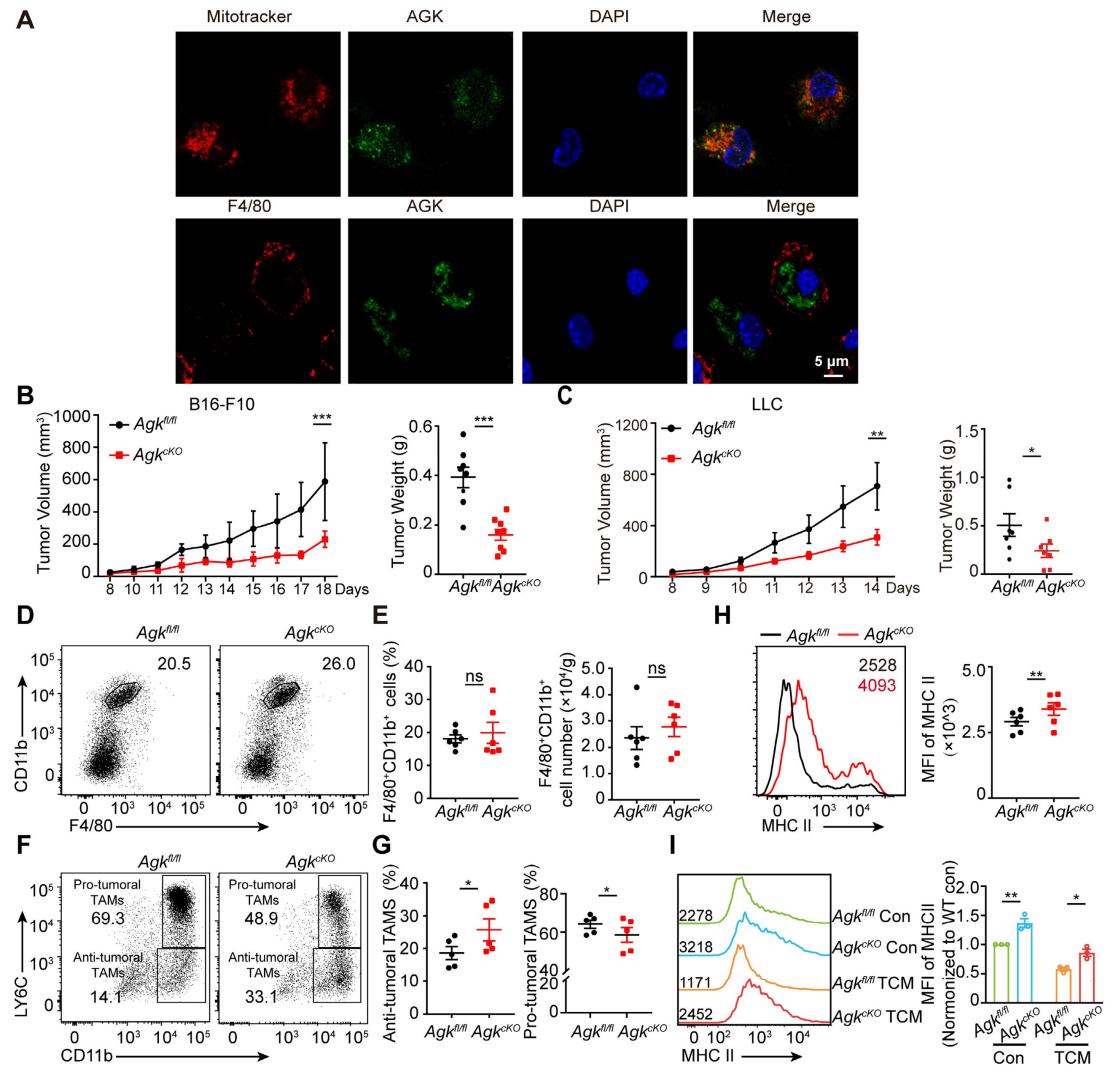
** Deletion of AGK in macrophages leads to enhanced anti-tumor activity.** (**A**) Mitochondrion (red) /AGK (Green) and F4/80 (Red) / AGK (Green) double immunostaining in BMDMs isolated from WT mice. (**B, C**) 5 × 10^5^ Lewis lung carcinoma cells (**B**) or 2 × 10^5^ melanoma B16-F10 cells (**C**) were subcutaneously injected into* Agk^fl/fl^* and *Agk^cKO^* mice, and the tumor volumes and weights were measured at the indicated times. (**D, E**) Flow cytometric analysis of CD11b^+^F4/80^+^ TAMs **(D)** and the number and percentages of the F4/80^+^CD11b^+^ TAMs (**E**) within LLC tumor tissues from *Agk^fl/fl^* and *Agk^cKO^* mice (n = 6 per group) were quantified. (**F, G**) Flow cytometric analysis of anti-tumoral TAMs (CD11b^+^LY6C^low^) and pro-tumoral TAMs (CD11b^+^LY6C^high^) (**F**) and the percentages of the anti-tumoral TAMs and pro-tumoral TAMs (**G**) within LLC tumor tissues from *Agk^fl/fl^* and *Agk^cKO^* mice (n = 5 per group) were quantified. (**H**) The mean fluorescence intensity (MFI) for MHC II on TAMs isolated from LLC- bearing *Agk^fl/fl^* and *Agk^cKO^* mice. (**I**) The mean fluorescence intensity (MFI) for MHC II expression on *Agk^fl/fl^* and *Agk^cKO^* BMDMs with or without the treatment of LLC-TCM (LLC-CM:DMEM = 1:3) for 24 h. Data were shown as mean ± SEM. and were analyzed by two-way ANOVA (B, C) or unpaired two-tailed t-test (E, G, H, I). Data in (A-I) are representative of one (A) or two (F) or three (B-E, H, I) independent experiments; ns, no significance; * p < 0.05; ** p < 0.01; *** p < 0.001.

To investigate the potential function of AGK in macrophages, we first evaluated the localization of AGK by co-staining of AGK with mitochondria marker MitoTracker Deep Red and membrane protein F4/80 in BMDMs. In contrast to both membrane and mitochondrial localization in T cells 18, AGK was co-localized with MitoTracker Deep Red but not with F4/80 in BMDMs (Figure 2A). Next, to address the role of AGK in macrophages in tumor development, we crossed *Agk^fl/fl^* mice with lysozyme-Cre (*Lyz^Cre^*) mice to generate *Agk^fl/^fl;Lyz^Cre^* mice (hereafter, *Agk^cKO^*; Figure S1A-B).

To investigate the function of macrophage AGK, we subcutaneously inoculated B16-F10 melanoma cells into *Agk^fl/fl^* and *Agk^cKO^* mice. After 2~3 weeks, tumor volumes and tumor weights in *Agk^cKO^* mice were significantly reduced, compared to those in *Agk^fl/fl^* mice (Figure 2B), indicating macrophage AGK may suppress macrophage anti-tumor activity *in vivo*. We observed similar results with another cancer cell line, LLC lung carcinoma cells (Figure 2C). In line with this, TUNEL and Ki-67 staining of the tumor tissues in the LLC tumor models showed enhanced apoptosis and reduced proliferation in *Agk^cKO^* mice (Figure S1C). In addition, the expression of both α-SMA and CD31, markers for vascular smooth muscle cells and endothelial cell generation respectively, were significantly reduced in LLC tumor tissues from *Agk^cKO^* mice (Figure S1D). However, the percentages and numbers of F4/80^+^CD11b^+^ TAMs within the LLC tumor tissues were comparable between *Agk^fl/fl^* and *Agk^cKO^* mice (Figure 2D, E). There was no difference of percentages of M1 (F4/80^+^CD11b^+^ CD11C^+^CD206^-^) or M2 (F4/80^+^CD11b^+^ CD11C^-^CD206^+^) macrophages within the tumors (Figure S1E-F).

To investigate whether AGK affects the macrophage maturation phenotype, we compared the expression of Ly6C and MHC-II in TAMs isolated from *Agk^fl/fl^* and *Agk^cko^* mice. We found that *Agk^cKO^* mice had a greater proportion of CD11b^+^Ly6C^low^ anti-tumoral TAMs but fewer CD11b^+^Ly6C^high^ pro-tumoral TAMs (Figure 2F, G). We detected the expression levels of MHC-II of TAMs in *Agk^fl/fl^* and *Agk^cKO^* LLC tumor tissues and found AGK deficiency promoted the expression of MHC-II (Figure 2H), indicating AGK could suppress the transition of TAMs into the anti-tumor phenotype. To further substantiate this finding, we isolated supernatants from LLC cells (1 × 10^6^) that were cultured in 10-cm plates with DMEM complete media for 5 days (hereafter, LLC-TCM) and stimulated *Agk^fl/fl^* and *Agk^cKO^* BMDMs with LLC-TCM for 24 h. We found that the expression of MHC-II was higher in *Agk^cKO^* BMDMs compared with wild type BMDMs (Figure 2I). Together, these data demonstrated that AGK in macrophages suppresses the maturation of TAMs and host anti-tumor activity *in vivo*.

### Deficiency of AGK in macrophages enhances T cell anti-tumor response

**Figure 3:**
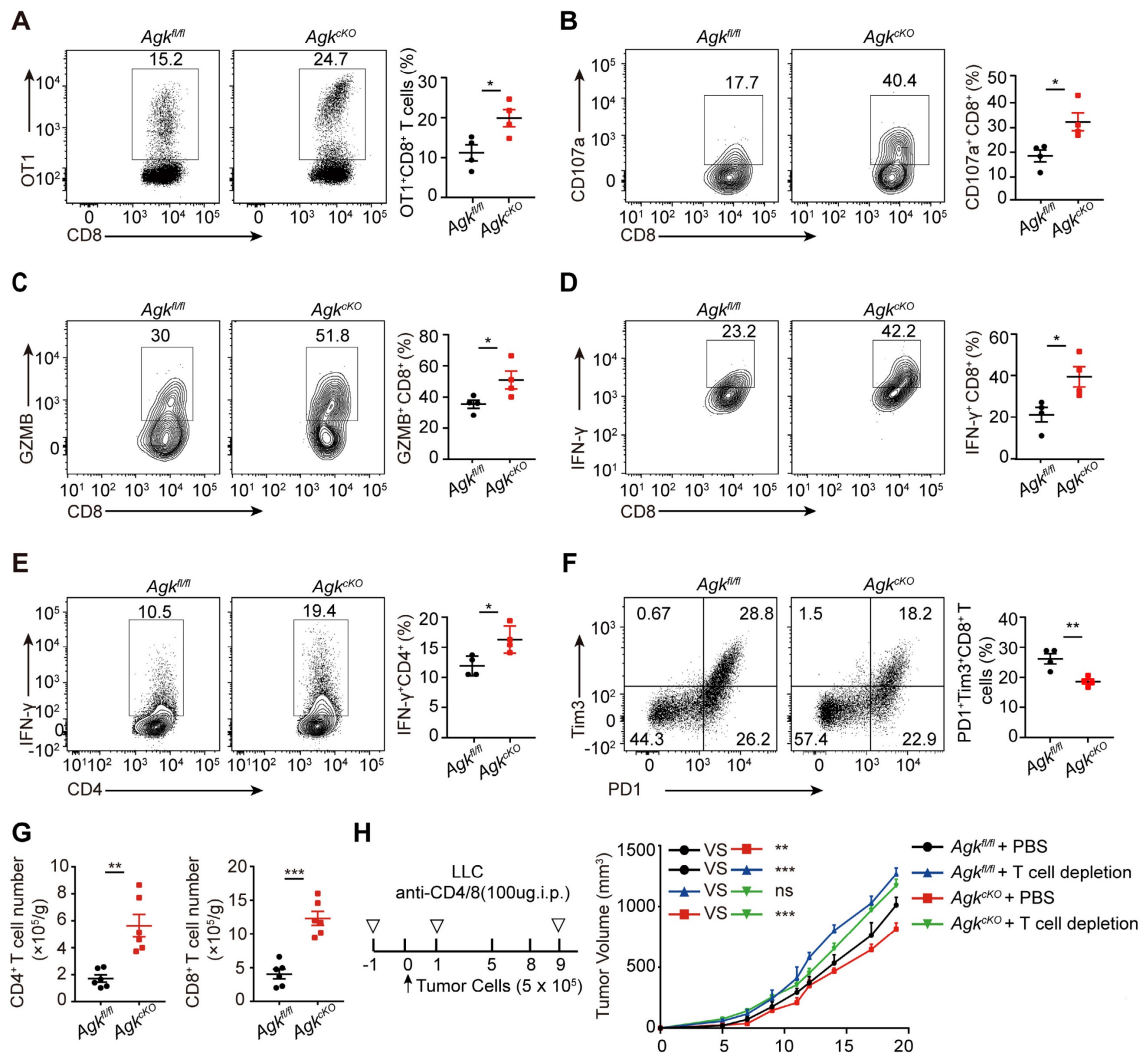
** Deficiency of AGK in macrophages enhances T cell anti-tumor response.** (**A**) *Agk^fl/fl^* mice or *Agk^cKO^* mice were inoculated *s.c.* with 5 × 10^5^ B16F10-OVA tumor cells and received *i.v.* adoptive transfer of 5 × 10^6^ activated OT-I CD8^+^ T cells on day 5, and the tumor were sacrificed at day 18. The percentages of OT-1 CD8^+^ T cells from *Agk^fl/fl^* and *Agk^cKO^* B16-OVA melanoma-bearing mice were quantified by flow cytometry. (**B-F**) The percentages of tumor-infiltrating IFN-γ^+^, GZMB^+^, CD107a^+^ cytotoxic CD8^+^ T cells and CD4^+^ Th cells, exhausted cell (PD1^+^Tim3^+^CD8^+^ T cells) population within the tumor tissues of B16-OVA bearing *Agk^fl/fl^* and *Agk^cKO^* mice were determined and quantified by flow cytometry (n = 4 per group). (**G**) Numbers of tumor-infiltrating CD4^+^ and CD8^+^ T cells from LLC*-*bearing* Agk^fl/fl^* and *Agk^cKO^* mice were detrmined by flow cytometry. (**H**) 5 × 10^5^ LLC cells were subcutaneously injected into* Agk^fl/fl^* and *Agk^cKO^* mice that were treated with PBS or anti-CD4/8 antibodies, and the tumor volumes were measured at the indicated times (n = 5 per group). Data were shown as mean ± SEM and were analyzed by two-way ANOVA (H) or unpaired two-tailed t-test (A-G). Data in A-H were representative of one (H) or two (A-F) or three (G) independent experiments. ns, no significance; * p < 0.05; ** p < 0.01; *** p < 0.001.

TAMs with high expression of MHC-II could directly maintain and promote functional activities of cytotoxic T cell 22. We first analyzed tumor-infiltrating T cells in *Agk^fl/fl^* and* Agk^cKO^* mice transplanted with B16-OVA melanoma cells and found that the percentages of tumor-specific OT-1 CD8^+^ T cells were higher in* Agk^cKO^* mice, compared to* Agk^fl/fl^* mice (Figure 3A). Furthermore, we observed higher percentages of tumor-infiltrating IFN-γ^+^ and GZMB^+^, CD107a^+^ cytotoxic CD8^+^ T cells and IFN-γ^+^ CD4^+^cells in *Agk^cKO^* mice compared with their counterparts in *Agk^fl/fl^* mice (Figure 3B-E). PD1^+^Tim3^+^CD8^+^ T cells are exhausted with impaired anti-tumor function 23. We detected the exhaustion status (PD1^+^Tim3^+^) of CD8^+^ tumor-infiltrating T cells in B16-OVA tumor-bearing mice and found that AGK deficiency significantly downregulated PD1 and TIM3 expression on CD8^+^ T cells (Figure 3F). Similarly, in the LLC model, flow cytometry and immunofluorescence results showed AGK deletion inhibited the infiltration of CD4^+^ T and CD8^+^ T cells and reduced the expression of inhibitory molecules *i.e.* PD1 and Tim3 on CD8^+^ T cells (Figure 3G; Figure S2B-D). By contrast, there was no significant difference of the CD4^+^Foxp3^+^ Treg percentages between *Agk^fl/fl^* and* Agk^cKO^* LLC tumor-bearing mice (Figure S2E). Depletion of T cells enhanced the tumor growth in both *Agk^fl/fl^* and* Agk^cKO^* LLC tumor-bearing mice and abolished any difference in tumor size between the two animal groups (Figure 3H; Figure S2F). Together, these data demonstrated that AGK-deficient macrophages inhibit tumor growth through their activation of T cells.

### AGK deficiency in macrophage enhances CD8^+^ T cell stemness

**Figure 4:**
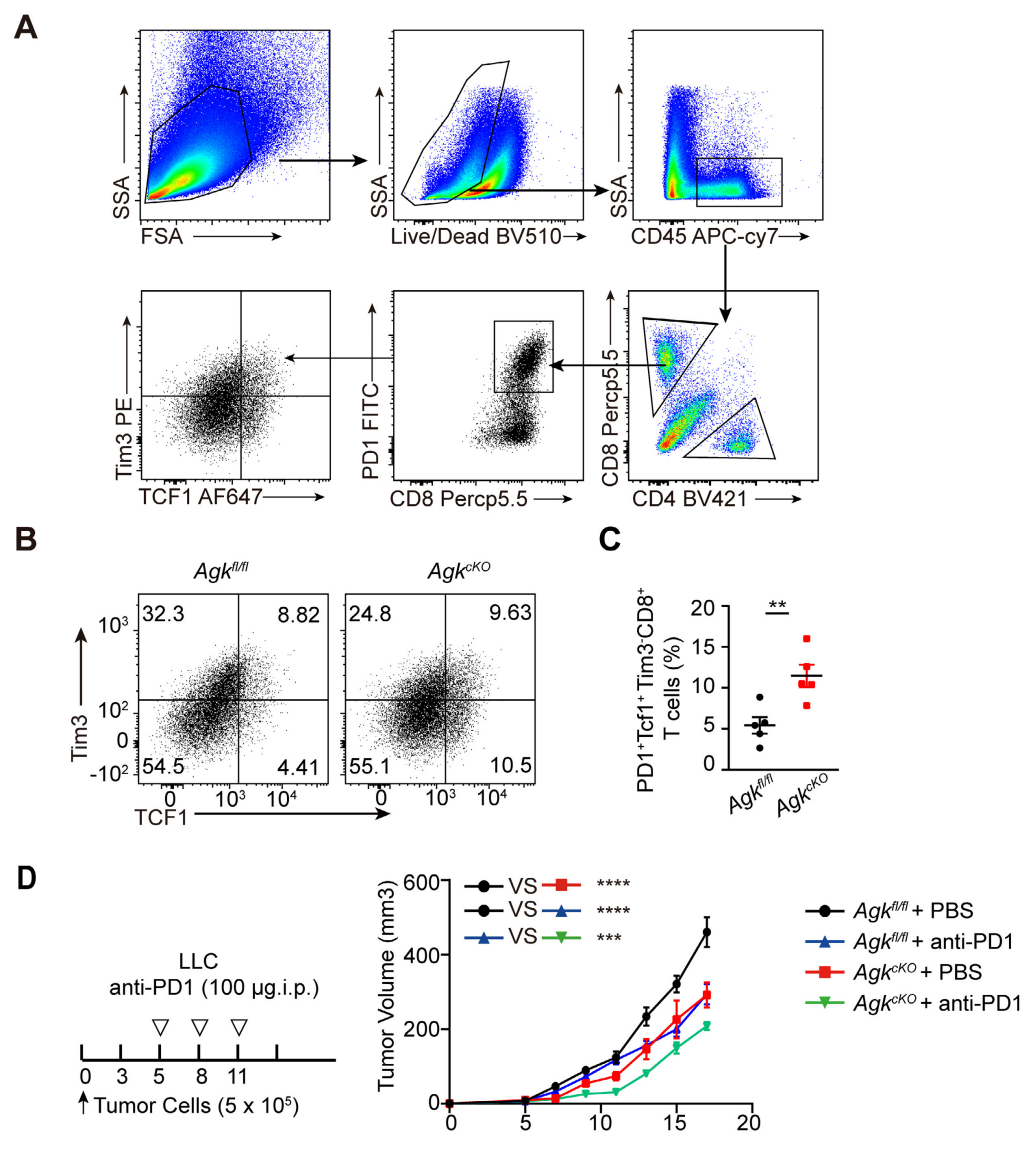
** AGK deficiency in macrophage enhances CD8^+^ T cell stemness.** (**A**) Flow gating strategy for stemness CD8^+^ T cells in *Agk^fl/fl^* or *Agk^cKO^* B16-OVA melanoma-bearing mice. (**B-C**) The percentages of stem-like (PD1^+^Tcf1^+^Tim3^-^CD8^+^ T cells) in tumor tissues of* Agk^fl/fl^* and *Agk^cKO^* B16-OVA melanoma-bearing mice were determined and quantified by flow cytometry (n = 5). (**D**) LLC cells were subcutaneously injected into* Agk^fl/fl^* and *Agk^cKO^* mice that were treated with PBS or anti-PD1 antibodies at day 5, 8 and 11, and the tumor volumes were measured at the indicated times (n *≥* 6 per group). Data were shown as mean ± SEM. and were analyzed by unpaired two-tailed t-test (**C**) and two-way ANOVA (**D**). Data in (B**-**D) were representative of two independent experiments. * p < 0.05; ** p < 0.01. *** p < 0.001.

Tcf^+^Tim3^-^ CD8^+^ T cells are known as 'precursor exhausted' T (Tpex) cells, which are stem cell-like CD8^+^ T cells with the capability to self-renew and generate more differentiated T cells and are critical for anti-tumor responses 24. We next detected the abundance of tumor-infiltrating stem-like CD8^+^ T cells in a B16-OVA tumor-bearing model (Figure 4A). We found that AGK deficiency significantly enhanced the percentages of stem-like PD1^+^Tcf1^+^Tim3^-^ CD8^+^ T cells in both B16-OVA (Figure 4B, C) and LLC models (Figure S3A, B). Tpex cells are reported as a predictive biomarker for a favorable clinical outcome of checkpoint therapy 25. To investigate whether the enhanced stem-like CD8^+^ T cells in *Agk^cKO^* mice could enhance anti-PD1/PD-L1 directed immunotherapy, we treated *Agk^fl/fl^* and* Agk^cKO^* LLC tumor-bearing mice with anti-PD1 antibody at day 5, day 8, and day 10 after tumor transplantation. The results showed *Agk^cKO^* mice further inhibited LLC tumor growth upon anti-PD1 treatment (Figure 4D). Together, these data demonstrated that AGK in macrophages regulated CD8^+^ T cell stemness and *Agk*-deficient macrophages can enhance the immunotherapies of PD1 blockade.

### Macrophage AGK deficiency promotes cGAS-STING signaling pathway and type I IFN response

**Figure 5:**
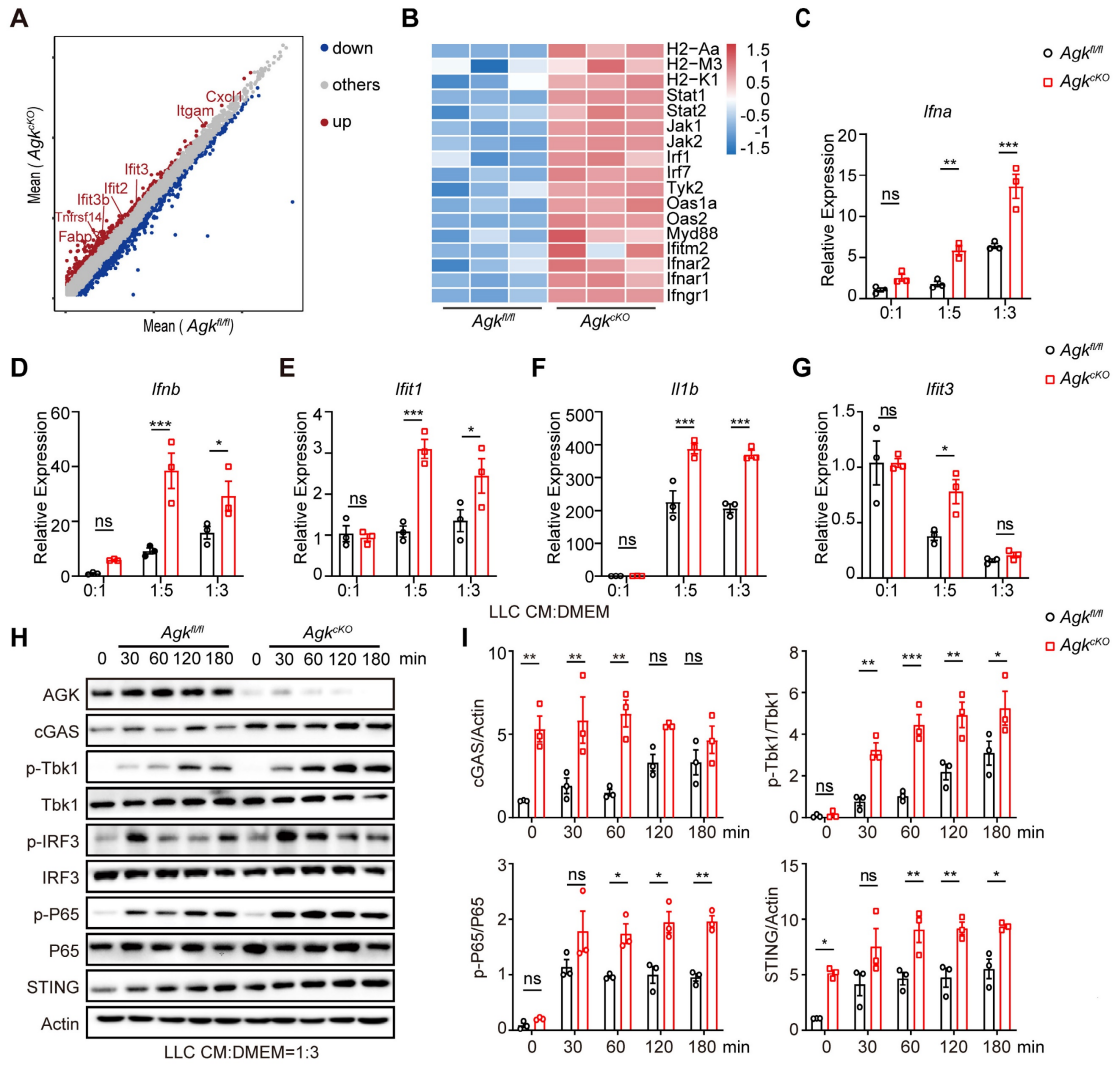
** Macrophage AGK deficiency promotes cGAS-STING signaling pathway and type I IFN response.** (**A-B**) RNA-Seq analysis of magnetic enriched CD11b^+^ TAMs isolated from *Agk^fl/fl^* or *Agk^cKO^* LLC tumor-bearing mice. (**A**) The volcano plot for differentially expressed genes between *Agk^fl/fl^* or *Agk^cKO^* TAMs (n = 3 per group, FC > 0.5). (**B**) Heatmap showing the expression patterns of genes between *Agk^fl/fl^* or *Agk^cKO^* TAMs, the color density indicates the expression levels of genes, each row was scaled by z-score. (**C-G**) The expressions of Type I IFN related and *Isgs* genes of* Agk^fl/fl^* or *Agk^cKO^* BMDMs upon treatment with LLC-TCM (LLC-CM:DMEM = 0:1; 1:5; 1:3) for 6 h. (**H, I**) Immunoblot analysis of (AGK, cGAS, p-Tbk1, Tbk1, p-IRF3, IRF3, p-P65, P65 and STING) in *Agk^fl/fl^* and *Agk^cKO^* BMDMs treated with LLC-TCM (LLC-CM:DMEM = 1:3) for 0, 30, 60, 120 and 180 min (**H**) and the band intensities were quantified (**I**). Data were shown as means ± SEM and were analyzed by two-way ANOVA (C-G, I). Data were representative of three experiments. ns, no significance; * p < 0.05; ** p < 0.01, *** p < 0.001.

To delineate the mechanism of how AGK deficiency in macrophages promoted T cell function, RNA-Seq analysis of TAMs from LLC tumor tissues in *Agk^fl/fl^* or *Agk^cKO^* mice was performed. We found 864 genes were downregulated and 1459 genes upregulated in the *Agk^cKO^* TAMs (Figure 5A). Among the upregulated genes, the antigen-presentation and type I IFN response related genes such as *Ifnar1*,* Ifnar2*, *Stat1*, and *Isgs* (*Ifit3* and *Ifitm2)* were significantly enriched in AGK-deficient TAMs (Figure 5B), indicating an enhanced type I IFN response in the *Agk^cKO^* TAMs compared with control macrophages. Next, we stimulated *Agk^fl/fl^* and *Agk^cKO^* BMDMs with different amounts of LLC-TCM and found that the expression of *Ifna*, *Ifnb, Ifit1, IL1b* and *Ifit3* genes was significantly higher in *Agk^cKO^* BMDMs compared with control macrophages (Figure 5C-G). We obtained similar results with B16 F10-TCM stimulated macrophages (Figure S4A-F).

Next, we compared the effect of AGK on activation of the cGAS-STING signaling pathway, an important regulator of type I IFN activation. LLC-TCM induced a significantly higher expression of cGAS and STING, enhanced prolonged phosphorylation of p65 and Tbk1, and enhanced transient phosphorylation of IRF3 in *Agk^cKO^* BMDMs compared with control macrophages (Figure 5H, I). B16-F10-derived TCM stimulation showed similar results on the activation of cGAS-STING pathway in the absence of AGK (Figure S4G). Together, these data demonstrated that AGK deficiency in macrophages enhanced the cGAS-STING signaling pathway and Type I IFN response.

### Deficiency of AGK in macrophages induces mitochondrial ROS production and mtDNA release

**Figure 6:**
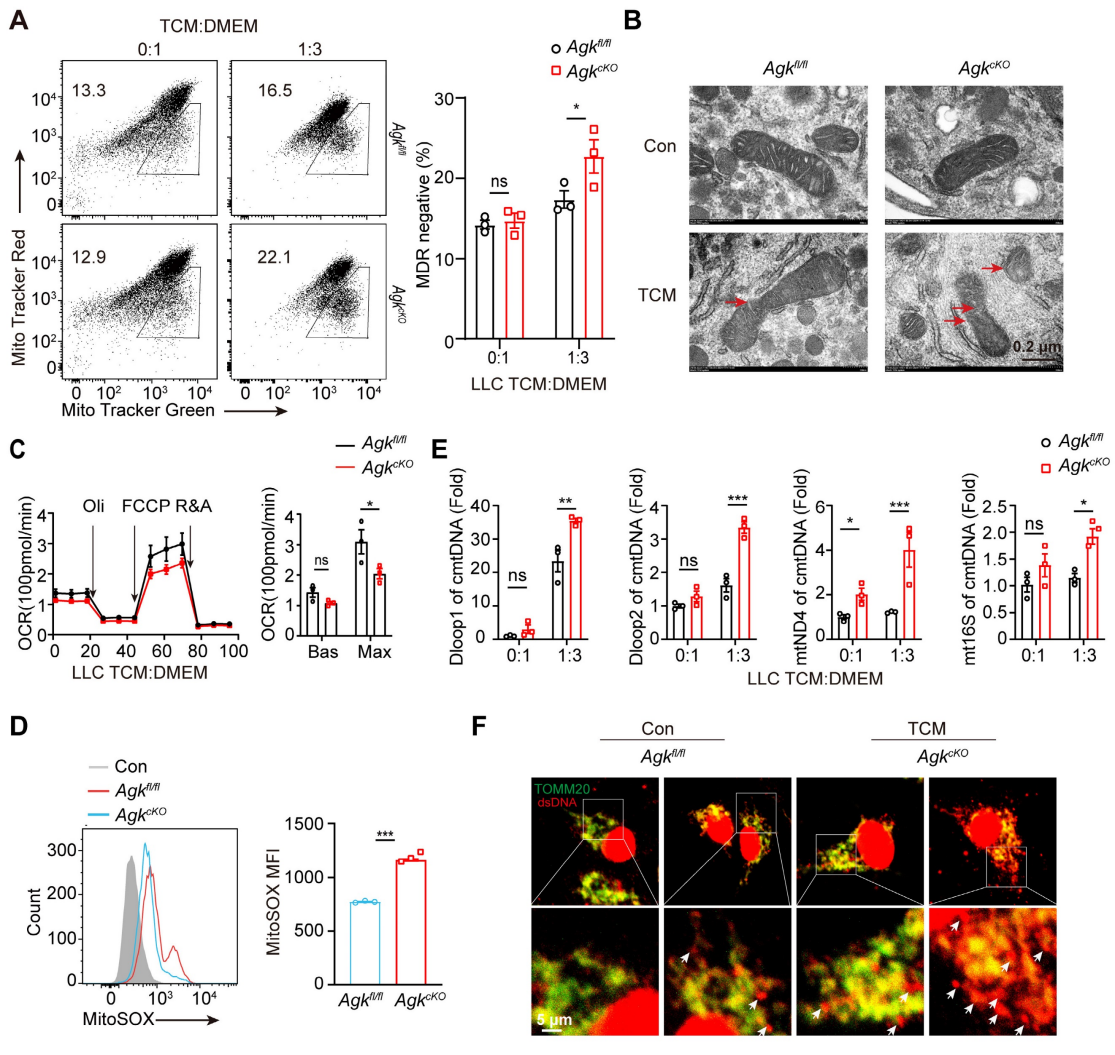
** Deficiency of AGK in macrophages induces mitochondrial ROS production and mtDNA release.** (**A-F**) *Agk^fl/fl^* and *Agk^cKO^* BMDMs were cultured with normal medium or LLC-TCM (LLC-CM:DMEM = 1:3) for 24 h. Cells were stained with MitoTracker Green and MitoTrack Deep Red (**A**) or imaged under electron microscope (**B**). (**C**) Extracellular flux analysis of the oxygen consumption rates (OCRs) stimulated for 24 h with LLC CM under basal conditions (Bas) or at maximum (Max) with the addition of oligomycin (Oli), the mitochondrial uncoupler carbonyl cyanide-4-(trifluoromethoxy) phenylhydrazone (FCCP), and rotenone plus antimycin A (R+A) (**D, E**) Cells were stained for MitoSOX (**D**) and the expressions of cytoplasmatic mtDNA (cmtDNA) of *Agk^fl/fl^* or *Agk^cKO^* BMDMs were quantified (**E**). (**F**) Representative immunofluorescence staining of mitochondrial TOMM20 (Green) and dsDNA (Red) in BMDMs from *Agk^fl/fl^* and *Agk^cKO^* mice. Data were shown as means ± SEM and were analyzed by two-tailed, unpaired Student's* t*-test (D) or two-way ANOVA (A, C). Data were representative of two (F) or three (A-D) experiments. ns, no significance; * p < 0.05; ** p < 0.01, *** p < 0.001.

Next, we investigated how the cGAS-STING pathway and type I IFN response were enhanced in *Agk^cKO^* macrophages. Released mtDNA has been shown as an important activator of the cGAS-STING pathway 26. We evaluated whether macrophage AGK deficiency could cause mitochondrial membrane damage and mtDNA release upon LLC-TCM treatment. The staining of MitoTracker Deep Red depends on the mitochondrial potential, while the staining of MitoTracker Green is not affected by the mitochondrial membrane potential, which represents the total mitochondria within living cells. The reduced rates of MitoTracker Deep Red positive cells in MitoTracker Green positive cells indicated the loss of mitochondrial potential. And we found that AGK-deficient macrophages had significantly reduced MitoTracker Deep Red staining (Figure 6A), indicating enhanced impairment of mitochondrial membrane integrity. While the mitochondrial morphology was similar in *Agk^fl/fl^* and *Agk^cKO^* BMDMs, LLC-TCM treatment induced more pronounced mitochondrial dysfunction in *Agk^cKO^* BMDMs, with more severe loss of the membrane structure and the disappearance of cristae under electron microscopy (Figure 6B). Furthermore, the OXPHOS rate of *Agk^cKO^* BMDMs was lower than *Agk^fl/fl^* BMDMs at maximum capacity (Figure 6C). The MitoSOX Red is a fluorogenic dye for selective detection of ROS in the mitochondria of live cells. Compared with *Agk*^fl/fl^ BMDMs, *Agk^cKO^* BMDMs had enhanced fluorescence intensity of MitoSOX staining (Figure 6D) and there was more accumulation of mtDNAs including D-loop 1, D-loop 2, mt16S, and mtND4 in the cytoplasm of *Agk^cKO^* macrophages upon LLC-TCM treatment (Figure 6E). To substantiate our qPCR data of mtDNA detection, immunofluorescence staining was performed using commercial antibodies against the common dsDNA and mitochondrial protein TOMM20. We found similar results showing that *Agk^cKO^* BMDMs released more dsDNA into cytoplasm than *Agk^fl/fl^* BMDMs under the treatment of LLC-TCM (Figure 6F). Together, these data demonstrated that AGK is required for the maintenance of mitochondrial integrity and limits the ROS generation and mtDNA release upon TCM stimulation.

### AGK-deficient macrophages promote the anti-tumor effects by inducing mitochondrial ROS and enhancing cGAS-STING-type I IFN response

**Figure 7:**
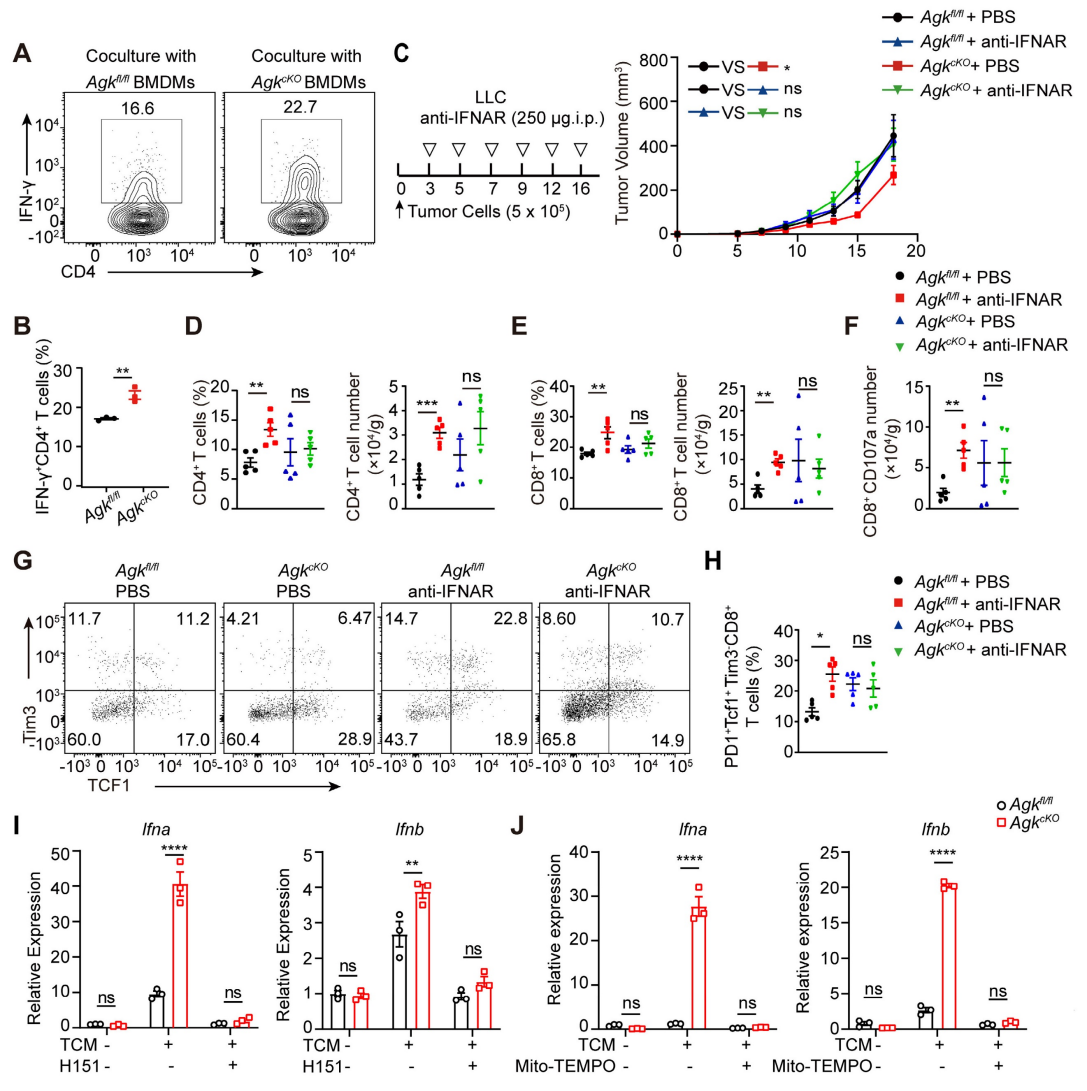
** AGK-deficient macrophages promote the anti-tumor effects by inducing mitochondrial ROS and enhancing cGAS-STING-type I IFN response.** (**A-B**) 2 × 10^5^ *Agk^fl/fl^* and *Agk^cKO^* BMDMs were pretreated with LLC-TCM (LLC CM: DMEM = 1:3) for 24 h and followed 6 h OVA 323-339 peptide-loaded. Then, cells were coclutured with 4 × 10^5^ OT-II CD4^+^T cells for 72 h. The IFN-γ expression in CD4 T cells was determined by flow cytometry. (**C**) 5 × 10^5^ LLC cells were subcutaneously injected into* Agk^fl/fl^* and *Agk^cKO^* mice that were treated with PBS or anti-IFNAR antibody i.p 250 μg at day 2, 4, 7 and 11, and the tumor volumes were measured at the indicated times (n *≥* 6 per group). (**D-H**) Flow cytometric analysis and the percentages and numbers of CD4^+^, CD8^+^, CD107a^+^CD8^+^ and PD1^+^Tcf1^+^Tim3^-^CD8^+^ T cells in four groups were quantified (n = 5). (**I**) The expressions of *Ifna* and* Ifnb* mRNAs in *Agk^fl/fl^* and *Agk^cKO^* BMDMs stimulated with LLC-TCM (LLC-CM:DMEM = 1:3) with or without H-151 (5 μM) for 6 h were determined by qRT-PCR. (**J**) *Agk^fl/fl^* and *Agk^cKO^* BMDMs were stimulated with LLC-TCM (LLC-CM:DMEM = 1:3) with or without Mito-TEMPO (500 μM) for 6 h. The expressions of *Ifna* and* Ifnb* mRNAs were determined by qRT-PCR. Data were shown as means ± SEM and were analyzed by two-way ANOVA (C) and unpaired two-tailed t-test (D-H) and were analyzed by two-way ANOVA (A, I, J). Data were representative of two (C-H) or three (A-B, I, J) experiments. ns, no significance; * p < 0.05; ** p < 0.01, *** p < 0.001.

We found that in the *Agk^cKO^* mice, the anti-tumor function of tumor-infiltrated CD8^+^ T cells was enhanced. To investigate the associated mechanism, we performed an *in vitro* antigen presentation assay by pulsing the *Agk^fl/fl^* and *Agk^cKO^* BMDMs with LLC-TCM and OVA 323-339 peptide. BMDMs pretreated with LLC-TCM and OVA 323-339 peptide were co-cultured with OT-II CD4^+^ T cells. Compared to *Agk^fl/fl^* BMDMs, *Agk^cKO^* BMDMs showed stronger ability to induce IFN-γ production by OT-II CD4^+^ T cells (Figure 7A-B), indicating deletion of AGK enhanced the macrophage antigen-presenting activity.

To further investigated whether AGK-deficient macrophages enhanced CD8^+^ T cell anti-tumor functions through type I IFN signaling, we blocked type I IFN signaling *in vivo* with anti-IFNAR antibody in the LLC tumor model. Blockade of type I IFN pathway led to enhanced tumor growth in *Agk^cKO^* mice, and there was no difference of tumor growth between *Agk^fl/fl^* and* Agk^cKO^* mice (Figure 7C), indicating the suppression of tumor growth in the *Agk^cKO^* mice is type I IFN dependent. In addition, blockade of IFNAR signaling eliminated the impact of AGK deficiency in macrophages on the percentages and numbers of tumor-infiltrating T cells (Figure 7D-E), CD107a^+^CD8^+^ T cells (Figure 7F; Figure S5A), and PD1^+^Tcf1^+^Tim3^-^CD8^+^ cells (Figure 7G-H). To determine whether the AGK-mediated inhibition of type I IFN response in macrophages is mediated by STING activation, we applied H-151, an antagonist of STING, to both *Agk^fl/fl^* and *Agk^cKO^* BMDMs in the absence or presence of LLC-TCM. Blockade of STING activation with H-151 significantly inhibited both *Ifna* and *Ifnb* expression in *Agk^cKO^* and *Agk^fl/fl^* BMDMs and there was no difference of *Ifna* and *Ifnb* expression in *Agk^cKO^* and *Agk^fl/fl^* BMDMs (Figure 7I), suggesting that AGK suppresses type I IFN responses in macrophages via cGAS-STING pathway. Similarly, we found that depletion of mitochondrial ROS with Mito-TEMPO, which is a superoxide dismutase mimetic that specifically scavenges mitochondrial ROS, also abolished the upregulation of *Ifna* and *Ifnb* in *Agk^cKO^* BMDMs (Figure 7J; Figure S5B).

These data suggested AGK-deficiency impaired the macrophages mitochondrial fitness, enhanced the generation of ROS and promoted the accumulation of mtDNA in cytoplasm, which subsequently activated cGAS-STING signals and type I IFN response.

## Discussion

The success of targeting inhibitor co-receptors including PD1 and CTLA4 in the treatment of cancers have highlighted the importance of the immune system in limiting tumor growth. Yet despite their success most cacner patients either do not benefit from PD1/PD-L1 inhibitors or soon relapse 27. In this study, we have identified AGK as an important regulator of TAM function. Our data demonstrate that the genetic deletion of AGK in macrophages enhanced their anti-tumoral capabilities by driving a type I IFN response that in turn increase anti-tumor T cell responses. Furthermore, AGK deficiency in macrophages improved the beneficial outcomes of anti-PD1 treatment by increasing the stemness of CD8^+^ T cells. AGK deletion led to increased production of mitochondrial ROS and the accumulation of mtDNA in the cytoplasm, which in turn triggered the activation of the cGAS-STING pathway and elicited type I interferon response. Notably, the deletion of AGK in macrophages enhanced the suppressive effect on tumor growth when combined with PD-1 blockade therapy.

AGK can promote the growth of human prostate cancer cell 28, breast cancer 21 and cervical squamous cell carcinoma 29. Here, we found AGK was mainly localized at the mitochondrial membrane in macrophages. The underlying mechanism that regulates the subcellular localization of AGK in immune cells is unknown. In our study, we found deletion of AGK led to enhanced mtDNA release and ROS production (Figure 6E-F). Similar with this, in liver cells deletion of AGK also resulted in accumulation of ROS in mitochondria through the disruption of interaction of AGK with mitochondrial respiratory chain complex I subunits, NDUFS2 and NDUFA10 30. Similarly, in our hand deletion of AGK impaired oxidative phosphorylation in macrophages while induced the production of mitochondrial ROS. Mitochondria are the primary organelles for ROS production and ROS is a major mediator for the accumulation of mtDNA in the cytoplasm 31. Treatment with Mito-TEMPO, a specific ROS scavenger of mitochondria, significantly decreased mtDNA level in primary hepatocytes cell supernatant 32, which is consistent with our study.

MtDNA released into the cytosol can promote systemic inflammation and is implicated in a variety of human diseases, including autoimmune, bacterial and viral infections, senescence and aging. For example, mtDNA and ox-mtDNA have been reported to be associated with periodic fever syndromes, rheumatoid and inflammatory arthritis, and systemic lupus erythematosus 33. Compelling evidence has accumulated linking DNA damages with activation of anti-tumoral immunity. DNA damage response (DDR) or exogenous insults such as chemotherapeutic drugs, ionizing radiation can induce cytosolic DNA accumulation in tumor cells 34, 35, which activates cGAS-STING and induces type I IFN and ISGs. It has been reported that BAX and BAK macropores induce widespread mitochondrial outer membrane permeabilization, which is essential for the release of mtDNA into the cytosol during the process of cell senescence 36. The precise mechanisms by which AGK limits mitochondrial ROS production and mtDNA accumulation in macrophages remain unclear and warrant further investigation.

Type I interferon (IFN) signaling characterized by expression of IFN-stimulated genes (ISGs) is critical for anti-tumor immunity by acting on both tumor cells and immune cells 37. Studies have demonstrated that increased secretion of IFN-β promotes the maturation of dendritic cells (DCs) and tumor-associated macrophages (TAMs), enhancing their antigen presentation capabilities and stimulating the activation of CD8^+^ T cells 38. In addition, type I IFN produced by intra-tumoral monocytes can regulate the polarization of TAMs and promote the crosstalk between natural killer (NK) cells and DCs, thus improving the efficacy of immune checkpoint blockade in melanoma 39-41. CD11b^+^ DCs have been shown to be the major source of IFN-β in multiple animal models, which depends on the cGAS-STING activation 42, 43. However, in a pre-established B16 tumors model, TAMs were the major source of IFN-β upon treatment of STING agonists (ML-RR-S2 CDA and DMXAA) compared to DCs, T cells, and tumor cells 44. These STING agonists also reprogram pro-tumor macrophages into an anti-tumor state, suggesting the potential of re-programing TAMs in regression of established tumors 45. Similarly, biologicals such as CD40 agonist antibodies or small molecules such as TLR agonist enhanced MHC-II^hi^ proinflammatory TAMs differentiation and enhanced CD8^+^ T cell anti-tumor activity 45-47. Class II HDAC inhibitors can also selectively reprogram monocytes and macrophages within the tumors, which leads to robust anti-tumor responses 11. In contrast, here we found that AGK acts as an intrinsic gatekeeper for mitochondrial integrity of macrophages and AGK deficiency promoted the ROS production and mtDNA accumulation, then activated cGAS-STING-type I IFN response. It remains unclear how AGK regulates the mitochondrial ROS production and mtDNA accumulation.

AGK is widely expressed in many cells and plays a vital role in physiological processes. Broad AGK inhibition may impair the physiological functioning of healthy cells other than macrophages. There are intensive efforts to specific targeting TAMs to enhance the efficacy of immunotherapies 48-50. Currently, several strategies have been developed to specific TAMs. Pun *et al*. reported a unique peptide sequence, M2pep identified by peptide library selection binds preferentially to TAMs 51. Macrophages express high levels of galactose-type lectin (Mgl) and Zhang* et al.* used galactosylated cationic dextran (gal-C-dextran), which can associate with CpG oligodeoxynucleotide (ODN) to form a stable nano-complex (GDO, gal-C-dextran+ODN), to specific deliver CpG ODN to TAMs 52. In addition, cholesteryl pullulan (CHP) nanogel particles with a diameter of less than 100 nm has been reported to target lymph node macrophages and thereby elicit the antigen presentation 53. With these and new upcoming approaches, it is possible to inhibit the AGK expression in TAMs but not affecting other cells.

Collectively, our research has unveiled a pivotal role for AGK in macrophages, as it contributes to the preservation of mitochondrial integrity and serves to limit the activation of the cGAS-STING pathway. Considering the pro-tumor effects of AGK in tumor cells and its ability to suppress the mtDNA-induced cGAS-STING pathway in macrophages, targeting AGK may offer a novel and promising strategy for enhancing immunotherapy in the context of cancer treatments.

## Method and Materials

### Mice

*Agk^fl/fl^* mice were generated by Dr Guihua Wang (HUST, Wuhan) OT-I and Lyz-Cre mice were kindly provided by Dr. Ning Wu (HUST, Wuhan) and OT-II mice were kindly provided by Dr. Huicheng Liu (HUST, Wuhan). *Agk^cKO^* mice were generated by crossing *Agk^fl/fl^* mice with Lyz-Cre mice. Wild type C57BL/6 mice were purchased from Beiente Company (Hubei, China). Six- to 12-week-old sex matched mice were used for experiments. Mice were housed in a specific pathogen-free facility with a 12-12 h light/dark cycle. All experiments were performed in accordance with the Institutional Animal Care and Use Committee of Tongji Medical College, HUST.

### Cell culture and collection of TCM

The mouse melanoma cell line B16-F10 and lung carcinoma LLC cells were obtained from ATCC. B16-OVA cells were provided by Dr. Weimin Wang (HUST, Wuhan). BMDMs were generated by culturing bone marrow cells of *Agk^fl/fl^* or *Agk^cKO^* mice in the presence of 20 ng/ml macrophage colony-stimulating factor (M-CSF) (catalog 315-02, PeproTech) for 5-7 days. All cell lines and bone marrow cells were cultured in RPMI DMEM (catalog C11995500BT, Gibco) supplemented with 10% FBS (catalog SA211.02, Cellmax), 1% penicillin streptomycin (catalog 15240062, Gibco). OT-I cells were separated from the spleens of 6-8-week-old OT-I mice using biotin-conjugated CD8 antibodies and Streptavidin MicroBeads (catalog 130-048-102, Miltenyi) andcultured in RPMI 1640 medium (catalog C11875500BT, Gibco) supplemented with 10% FBS (catalog 1715753, Gibco), 1% penicillin/streptomycin (catalog 15240062, Gibco), 1 mM pyruvate (catalog 11360070, Gibco), 1× NEAA (catalog 11140050, Gibco) and 50 μM β-mercaptoethanol (catalog m3148, Sigma) and were activated with 0.5 μg/ml anti-CD3 (catalog BE0001-1, BioXell) as well as 1 μM OVA 257264 peptide (catalog T510211, Sangon Biotech) for OT-I cells. After 2 days, cells were washed and resuspended in complete media containing 20 ng/ml recombinant human IL-2 (catalog 200-02-100, PeproTech) for 5 days. All cells were incubated at 37 °C with 5% CO2.

LLC cells and B16-F10 melanoma cells were cultured with RPMI full medium for 5 days. Cultured media were collected and centrifuged at 12000g for 5 min, followed by filtration with 0.2 μm filters.

### Syngeneic mouse models

2 × 10^5^ B16-F10 or 5 × 10^5^ LLC cells were injected *s.c.* into the flanks of 6- to 12-week- *Agk^fl/fl^* mice or *Agk^cKO^* mice. Tumor growth was measured every 2 to 3 day at 7-9 day post tumor inoculation using digital calipers. For B16-OVA model, *Agk^fl/fl^* mice or *Agk^cKO^* mice were inoculated *s.c.* with 5 × 10^5^ B16F10-OVA tumor cells and received *i.v.* adoptive transfer of 5 × 10^6^ activated OT-I CD8^+^ T cells on day 6. For T cell depletion, mice were injected *i.p.* with 200 μg of anti-CD4 (clone GK1.5, BioXcell) or anti-CD8 (clone 2.43, BioXcell) on the day before implantation, day 1 and day 7 after tumor implantation. For PD-1 depletion, mice were *injected i.p.* with 100 μg of anti-PD1 (clone RMP1-14, BioXcell) on day 5, 8 and 11 after tumor inoculation. For the blockade of Type I IFN *in vivo*, mice were injected i.p. with 250 μg of anti-IFNAR-1 (clone MAR1-5A3, BioXcell) on day 2, 4, 7 and 11 after tumor inoculation.

### TILs Isolation

Tumor tissues were dissected and gently minced and stirred into small pieces. Afterwards, tumor tissues were digested with 1 × PBS containing 200 ng/ml collagenase IV (catalog V900893, Invitrogen), 40 ng/ml DNase I (catalog 10104159001, Roche), 250 ng/ml hyaluronidase (catalog H1136, Sigma), at 37 °C for 60 min. Cell suspensions were filtered and red blood cells were lysed with ACK lysing buffer. Tumor-infiltrating lymphocytes were enriched by density gradient centrifugation against 40%/80% Percoll (catalog 17089102, GE Life). TILs were stained and analyzed by flow cytometry.

### Flow cytometry

For cell surface staining, TIL cells were first blocked with anti-CD16/CD32 Fc blockade (clone 2.4G2, Biolegend), then stained with the following antibodies: anti-CD45(clone 30-F11), anti-CD4 (clone RM4-5), anti-CD8 (clone 53-6.7), anti-CD44 (clone IM7), anti-F4/80 (clone BM8), anti-CD11b (clone M1/70), anti-CD11c (clone N418), anti-CD206 (clone C068C2), anti-TCRVα2 (clone B20.1), anti-PD1 (clone 29F.1A12), anti-TIM3 (clone RM73-2.3) (all from Biolegend).

For intracellular staining, cells were stimulated with 50 ng/ml PMA (catalog P1585, MilliporeSigma) and 500 ng/ml ionomycin (catalog I3909, MilliporeSigma), together with 5 μg/ml GolgiPlug (catalog 555029, BD) in complete RPMI 1640 medium for 4 h at 37 °C and stained with a Cytofix/Cytoperm Fixation/Permeabilization Kit (catalog 554714, BD). The following antibodies were used: anti-IFN-γ (clone XMG1.2, BD), anti-TNF-α (clone MP6-XT22, Biolegend), and anti-granzyme B (clone GB11, BD). For transcription factors staining, cells were stained with the Foxp3/Transcription Factor Staining Buffer Set (catalog 00-5523-00, eBioscience) according to the manufacturer's recommendations. The following antibodies were used: anti-Foxp3 (clone FJK-16s, Biolegend), anti-TCF-1 (clone C63D9, CST). Samples were collected on a BD FACS Verse flow cytometer or BD LSR Fortessa, and data were analyzed using FlowJo software (Tree Star).

### qRT-PCR

Cells were harvested and total RNAs were extracted by Trizol (catalog 15596026, Invitrogen) and then reverse transcribed to cDNA with cDNA Reverse Transcription Kit (catalog TSE203, TSINGKE) following the manufacturer's instructions. mRNA expression levels were analyzed using Bio-Rad SYBR Green intercalating fluorophore system (Applied Biosystems). Data were normalized to the expression of β-actin using ^ΔΔ^CT. The following primer sets were used, as listed in Table S1.

### Immunofluorescence

For the determination of AGK subcellular localization in BMDMs, 5 × 10^5^ cells were seeded on the Laser confocal culture dishes and cultured overnight at 37 ^o^C incubator for proper attachment. Then cells were treated with 1:10,000 PBS-diluted MitoTracker™ Deep Red FM (catalog M22426, Invitrogen) for 15 min. Afterwards, cells were washed twice with PBS and fixed with 4% paraformaldehyde for 10 min, and permeabilized with 0.5% Triton X-100 for 20 min. After blocking with 5% BSA for 1 h, cells were stained with anti-AGK (catalog NBP2-49085, Novus) and anti-F4/80 (clone BM8, Invitrogen) overnight at 4 °C. Secondary fluorescent antibodies (FITC, Cy3 from Life Technologies) were added for 1 h and DAPI was used for nuclear counterstaining. Samples were imaged through a confocal microscope (Olympus) 24 h after mounting.

### Immunohistochemistry

Tumor tissues were isolated and fixed in formalin, embedded in paraffin, and sectioned for staining with hematoxylin and eosin. Following antibodies were used: anti-Ki67 (GB151142, Servicebio), anti-BCL-2 (GB114830, Servicebio), anti-CD31 (GB13063, Servicebio), α-SMA (GB13044, Servicebio), anti-CD4 (GB15064, Servicebio), anti-CD8 (GB15068, Servicebio), TUNEL staining was performed flowing the manufacturer (GDP1041, Servicebio).

### Immunoblot analysis

Cells were lysed in RIPA lysis buffer (25 mM Tris-HCl, pH 7.6, 150 mM NaCl, 1% NP-40, 1% sodium deoxycholate, 0.1% SDS containing protease inhibitor cocktail and phosphatase inhibitor cocktail) for 1 h on ice. Protein concentrations were determined using a BCA Protein Assay Kit and loaded onto SDS-PAGE gels and transferred onto nitrocellulose membranes. The membranes were then incubated with primary antibodies including anti-AGK (catalog NBP2-49085, NOVUS), anti-cGAS (clone D3O8O, CST), anti-STING (clone D2P2F, CST), anti-phospho-TBK1/NAK (Ser172) (clone D52C2, CST), anti-TBK1/NAK (clone D1B4, CST), anti-phospho-IRF-3 (Ser396) (clone D6O1M, CST), anti-IRF-3 (clone D83B9, CST), anti-p-p65Ser536 (clone 93H1, CST), anti-p65 (clone D14E12, CST), anti-phospho-Stat1 (Tyr701) (clone 58D6, CST), anti-Stat1 (clone D1K9Y, CST), anti-actin (clone 13E5, CST). After the non-specific binding sites were blocked by 5% milk for 2 h, and primary antibodies incubated overnight at 4 ^o^C, appropriate secondary HRP-conjugated antibodies were followed, then all blots were developed with enhanced chemiluminescent (ECL) (GE healthcare) and analyzed by ImageJ software.

### Quantification of mtDNA release

1 × 10^6^ cells BMDMs were resuspended in 100 μl of digitonin buffer containing 50 mM HEPES pH 7.4,150 mM NaCl, and 25 μg/ml digitonin (catalog 11024-24-1, Millipore). The mixtures were incubated at room temperature on a rotator for 10 min, followed by centrifugated at 16,000 g for 25 min at 4 °C. The supernatant containing (cmtDNA) was used for q-PCR. For total mtDNA measurement, the pellets were mixed in 200 μl of lysis buffer containing 5 mM EDTA and proteinase K (catalog 19157, Qiagen), then incubated at 55 °C overnight. The digested pellets were heated at 95 °C for 20 min to inactivate proteinase K. DNAs were purified (catalog K0721, Thermo Fisher) and the samples were subjected to q-PCR with mtDNA specific primers (Table. S1). The cmtDNA was normalized to the total mtDNA in the pellet for each sample.

### RNA-sequencing analysis

TILs were isolated from LLC-bearing *Agk^fl/fl^* mice or *Agk^cKO^* mice (n = 3) at day 18. TAMs cells were enriched from TILs using biotin antibody anti-CD11b (clone M1/70, Biolegend) and anti-F4/80 (clone BM8, Biolegend) staining for 30 min, followed by a positive magnetic selection using streptavidin microbeads (catalog 130-048-102, Miltenyi). Total RNAs were isolated with TRIzol (catalog 15596026, Invitrogen) and subjected to RNA-sequencing using Illumina Nextseq 6000. Using the R package limma (46), differentially expressed genes were identified with a cutoff of log2(fold change) > 0.5.

### Antigen presentation assay

BMDMs from *Agk^fl/fl^* mice or *Agk^cKO^* mice were collected on day 6 and stimulated with LLC-TCM for 24 h. BMDMs were washed with PBS and stimulated with 10 μg/ml OVA 257-264 peptide (catalog T510211, Sangon Biotech) or OVA 323-339 peptide (catalog T510212, Sangon Biotech) for 6 h. After that, cells were washed once with PBS and co-cultured with naive OT-I CD8^+^ T cells or naïve OT-II CD4^+^ T cells from OT-I or OT-II mice for 3 days. PMA was added to the co-cultures 4 h before the end of incubation. The cross-priming capacity of macrophages was then evaluated by flow cytometry measuring the percentages of IFN-γ producing CD8^+^ T cells.

### Metabolism analysis

4 × 10^4^ *Agk^fl/fl^* or *Agk^cKO^* BMDMs cells per well were plated into 24-well seahorse assay plate and cultured overnight. Afterwards, BMDMs were stimulated with LLC-TCM for 24 h. Cells were washed twice with Seahorse XF DMEM medium (catalog 103575-100, Agilent Technologies). Cells were treated with either 2.5 μM Oligomycin and 2.5 μM FCCP and 0.5 μM Rotenone and Antimycin A for OCR testing or 10 mM glucose and 1 mM Oligomycin and 50 mM 2-DG for ECR testing. The ECAR and OCR for each well were determined by Seahorse XFe96 Extracellular Flux Analyzer.

### Analysis of data from TCGA

RNA sequencing data in The Cancer Genome Atlas (TCGA) is available online. Cancers without normal tissues were excluded. AGK expression data were extracted from the obtained data sets using R programming language (version 4.2.1). The ESTIMATE algorithm was used to estimate the levels of infiltrating stromal and immune cells through gene expression data. Stromal signature and Immune signature were designed to represent the infiltration of stroma and immune cells in tumor tissues. Gene expression profiles of normal hematopoietic samples were compared with those of other normal cell types, and the overlap between the two gene sets constituted the immune signature. Stromal-related genes were selected among non-hematopoiesis genes by comparison of the tumor cell fraction and matched stromal cell fraction in breast, colorectal and ovarian cancer data sets. Genes with high variability in cancer cell lines and genes highly expressed in glioma stem-like cells were filtered to make up the stromal signature. These two single-sample ssGSEA signatures were used to generate scores that reflect the presence of each cell type in tumour samples and combined represent a measurement of tumor purity 54. The method is publicly available through the SourceForge software repository 54. The boxplots were generated using the ggplot2 package (version 3.4.3) in the R programming language, and Wilcoxon tests were conducted for the purpose of differential analysis.

### Analysis of public scRNA-seq data

All public scRNAseq data were downloaded from the Gene Expression Omnibus database (LUAD: GSE229253, COAD: GSE146771 and OV: GSE180884). For each set of data, the steps of quality control, dimensionality reduction, clustering and cell annotation were performed and then the macrophaegs cells were extracted, expression of AGK in macrophages was compared between the normal and tumor groups.

### Statistical analysis

Statistical analysis was performed with GraphPad Prism 9. Statistical significance was determined by unpaired Student's t test or for variances by one-way ANOVA or two-way ANOVA. No statistically significant differences were considered when *p* values were more than 0.05. Data are presented as Means ± SEM.

### Data availability

Raw RNA sequencing data are available from the corresponding author upon reasonable request.

## Supplementary Material

Supplementary figures.

## Abbreviations

AGK: acylglycerol kinase
BMDMs: bone marrow derived macrophages
BRCA: breast cancer
BLCA: bladder urothelial cancer
COAD: colon adenocarcinoma
cGAS-STING: cyclic GMP-AMP and the cyclic GMP-AMP receptor stimulator of interferon genes
CHP: cholesteryl pullulan
DAMPs: damage-associated molecular patterns
DLBCL: diffuse large B cell lymphoma
DDR: DNA damage response
DCs: dendritic cells
ESCA: esophageal cancer
ISGs: IFN-stimulated genes
GTEx: genotype tissue expression
gal-C-dextran: galactosylated cationic dextran
LUSC: lung squamous cell cancer
LUAD: lung adenocarcinoma
Lyz-Cre: lysozyme-cre
NK: natural killer cells
Mgl: macrophage galactose-type lectin
ODN: oligodeoxynucleotide
PRAD: prostate adenocarcinoma
RNA-seq: rna sequencing
READ: rectum adenocarcinoma
STAD: stomach adenocarcinoma
TAMs: tumor associated macrophages
mtDNA: mitochondrial DNA
Tpex: precursor exhausted T cells
TME: tumor microenvironment
mTAMs: mature TAMs
iTAMs: immature TAMs
TIM22: mitochondrial inner membrane 22
TCGA: the cancer genome atlas
