# Supplementary material for: Acylglycerol kinase inhibits macrophage anti-tumor activity via limiting mtDNA release and cGAS-STING-type I IFN response
Source: Theranostics. 2025 Jan 1;15(4):1304–19. doi: 10.7150/thno.101298 (PMC11729555; doi:10.7150/thno.101298)
Supplement: Supplementary file 1 — Supplementary figures. [file thnov15p1304s1.pdf]

Figure S1. Deletion of AGK in macrophages leads to enhanced anti-tumor activity

(A, B) qRT-PCR and immunoblotting analysis of the expression of AGK in $Agk^{fl/fl}$ or Agk^{cKO} macrophages (n = 3 per group). (**C)** H&E and TUNEL, Ki67 staining of tumor tissues from LLC-bearing $Agk^{fl/fl}$ and Agk^{cKO} mice. (**D)** CD31 (Red) /α-SMA (Green) double immunostaining of tumor tissues from LLC-bearing $Agk^{fl/fl}$ and Agk^{cKO} mice. (**E-F)** The percentages of M1 ($CD11C^{+}CD206^{-}$) or M2 ($CD11C^{-}CD206^{+}$) among gated $F4/80^{+}CD11b^{+}$ macrophages in tumor tissues from LLC-bearing $Agk^{fl/fl}$ and Agk^{cKO} mice were determined and quantified by flow cytometry (n = 5). Data were shown as mean ±SEM and were analyzed by unpaired two-tailed t-test (A, D, F). Data in (A-F) are representative of three independent experiments; ns, no significance; *$p < 0.05$, ** $p < 0.01$, *** $p < 0.001$.

Figure S2. Deficiency of AGK in macrophages enhances T cell anti-tumor response

(**A**) Flow gating strategy for cytokines and cell surface staining in $CD8^{+}$ T cells in $Agk^{fl/fl}$ or Agk^{cKO} B16-OVA melanoma-bearing mice. (**B, C**) Representative percentages and immunofluorescence staining of $CD4^{+}$ T cell and $CD8^{+}$ T cell in LLC tumor tissues from $Agk^{fl/fl}$ and Agk^{cKO} tumor mice (n ≥ 5 per group). (**D**) Representative plots and percentages of $PD1^{+}Tim3^{+}CD8^{+}$ T cells in the tumor tissues from $Agk^{fl/fl}$ and Agk^{cKO} LLC-bearing mice (n ≥ 4 per group). (**E)** Representative plots and percentages of $CD4^{+}$ $Foxp3^{+}$ T cells population. (**F)** Representative plots of $CD4^{+}$ T cells and $CD8^{+}$ T cells isolated from LLC tissues of $Agk^{fl/fl}$ and Agk^{cKO} mice treated with PBS or anti-CD4/8 antibody (n ≥ 3 per group). Data were means ± SEM and were analyzed by two-tailed, unpaired Student's *t*-test (B-E). Data were representative of one (E, F) or three (B, D) experiments. ns, no significance; * $p < 0.05$; ** $p < 0.01$, *** $p < 0.001$.

Figure S3. AGK deficiency in macrophage enhances $CD8^{+}$ T cell stemness

(**A, B**) Representative plots and percentages of $PD1^{+}Tcf1^{+}Tim3^{-}CD8^{+}$ T cells population determined by flow cytometry in LLC-bearing $Agk^{fl/fl}$ and Agk^{cKO} mice (n = 6 per group). Data were shown as means ± SEM and were analyzed by two-tailed, unpaired Student's *t*-test (B). Data were representative of two (A, B) experiments. ns, no significance; * $p < 0.05$; ** $p < 0.01$,*** $p < 0.001$.

Figure S4. Macrophage AGK deficiency promotes cGAS-STING signaling pathway and type I IFN response

(A-F) The expressions of Type I IFN related genes were determined by RT-PCR in $Agk^{fl/fl}$ and Agk^{cKO} BMDMs upon treatment with B16-TCM (B16 CM:DMEM = 0：1; 1：5; 1：3) for 6 h. (**G**) Immunoblot analysis of the amounts of (AGK, cGAS, p-Tbk1, Tbk1, p-IRF3, IRF3, p-P65, P65 and STING) in $Agk^{fl/fl}$ and Agk^{cKO} BMDMs that were treated with B16F10-TCM (LLC CM:DMEM = 1：3) for 0, 30, 60, 120 and 180 min. Data were means ± SEM and were analyzed by and two-way ANOVA (A-F). Data were representative of two (G) or three (A-F) experiments. ns, no significance; * $p < 0.05$;** p < 0.01; *** p < 0.001.

Figure S5. AGK-deficient macrophages promote the anti-tumor effects by inducing mitochondrial ROS and enhancing cGAS-STING-type I IFN response

(A) 5×10^{5} LLC cells were subcutaneously injected into $Agk^{fl/fl}$ and Agk^{cKO} mice that were treated with PBS or anti-IFNAR antibody i.p 250 μg at day 2, 4, 7 and 11 and the tumor volumes were measured at the indicated times (n = 5 per group). Flow cytometric analysis and the percentages of $CD107a^{+}CD8^{+}$ T cells in four groups were quantified.

(**B**) *Agk*$^{fl/fl}$ or *Agk*^{cKO} BMDMs were cultured with or without Mito-TEMPO (500μM) for 24 h and MitoSOX levels were determined by flow cytometry. Data were means ± SEM and were analyzed by two-tailed, unpaired Student's *t*-test (A) or two-way ANOVA (B). Data were representative of one (A) or three (B) experiments. ns, no significance; * $p < 0.05$; ** $p < 0.01$; *** $p < 0.001$.

Figure S1

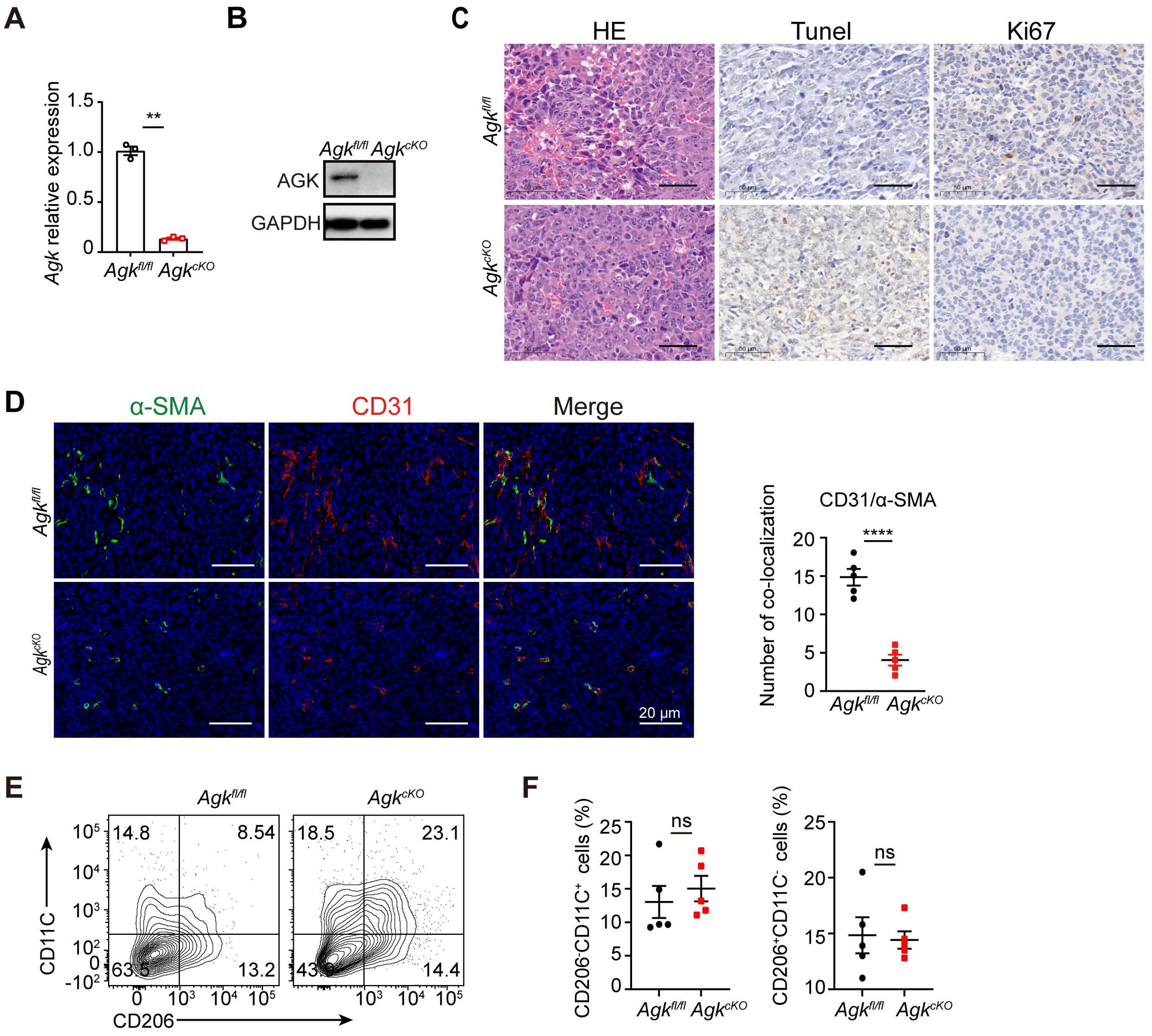
A
Agk relative expression
1.5
1.0
0.5
0
**
Agk^fl/fl
Agk^cKO
B
Agk^fl/fl
Agk^cKO
AGK
GAPDH
C
HE
Tunel
Ki67
Agk^fl/fl
Agk^cKO
D
α-SMA
CD31
Merge
Agk^fl/fl
Agk^cKO
20 μm
CD31/α-SMA
Number of co-localization
20
15
10
5
0
****
Agk^fl/fl
Agk^cKO
E
Agk^fl/fl
Agk^cKO
CD11C
CD206
14.8
8.54
63.5
13.2
18.5
23.1
43.9
14.4
F
CD206-CD11C+ cells (%)
25
20
15
10
5
0
ns
Agk^fl/fl
Agk^cKO
CD206+CD11C- cells (%)
25
20
15
10
ns
Agk^fl/fl
Agk^cKO

Figure S2

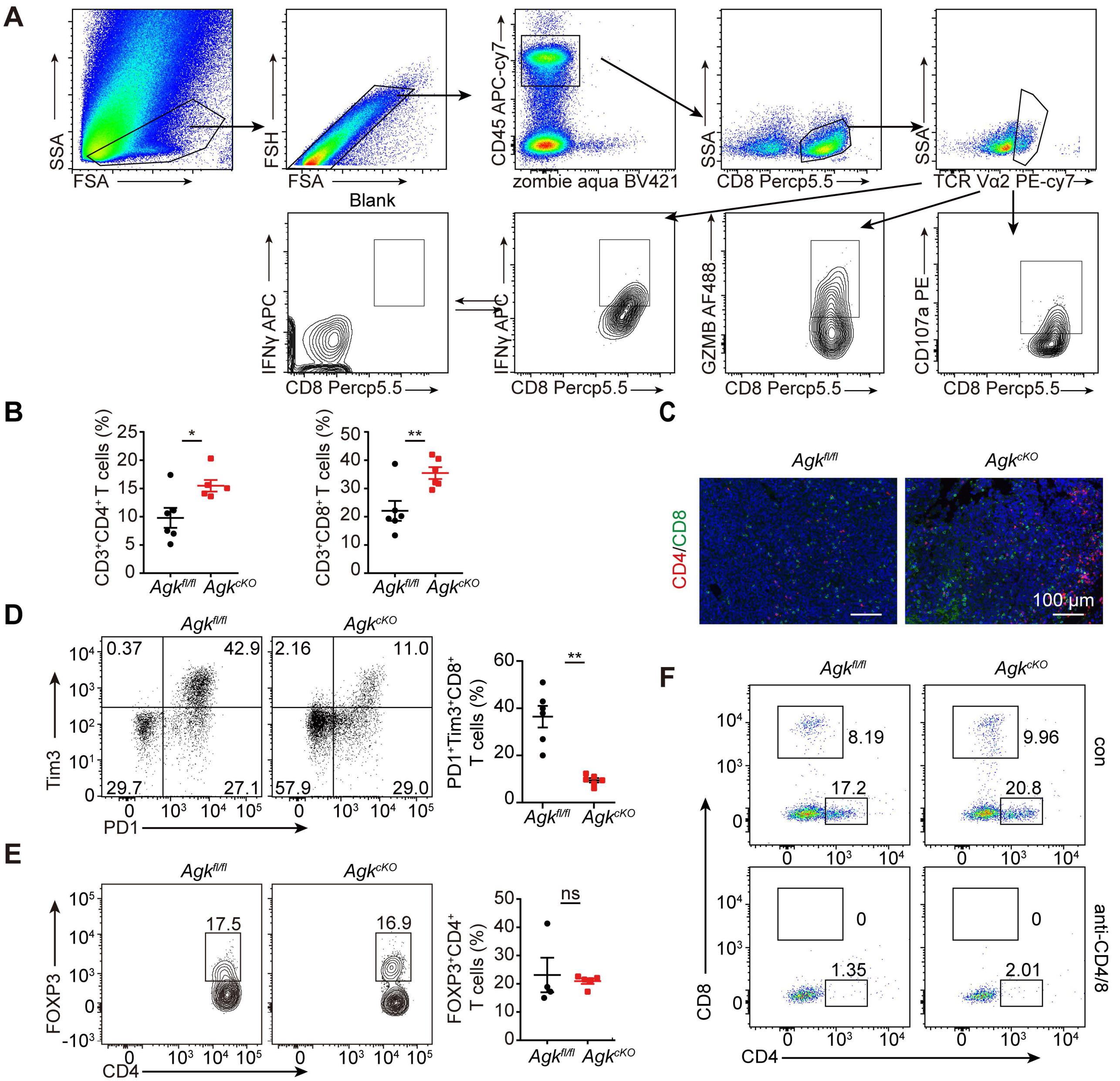

A
SSA
FSA
FSH
FSA
Blank
CD45 APC-cy7
zombie aqua BV421
SSA
CD8 Percp5.5
SSA
TCR Vα2 PE-cy7
IFNγ APC
CD8 Percp5.5
IFNγ APC
CD8 Percp5.5
GZMB AF488
CD8 Percp5.5
CD107a PE
CD8 Percp5.5
B
CD3⁺CD4⁺ T cells (%)
*
Agk^fl/fl
Agk^cKO
CD3⁺CD8⁺ T cells (%)
**
Agk^fl/fl
Agk^cKO
C
Agk^fl/fl
Agk^cKO
CD4/CD8
100 μm
D
Agk^fl/fl
Agk^cKO
0.37
42.9
29.7
27.1
2.16
11.0
57.9
29.0
Tim3
PD1
PD1⁺Tim3⁺CD8⁺ T cells (%)
**
Agk^fl/fl
Agk^cKO
E
Agk^fl/fl
Agk^cKO
17.5
16.9
FOXP3
CD4
FOXP3⁺CD4⁺ T cells (%)
ns
Agk^fl/fl
Agk^cKO
F
Agk^fl/fl
Agk^cKO
8.19
17.2
9.96
20.8
con
0
1.35
0
2.01
anti-CD4/8
CD8
CD4

Figure S3

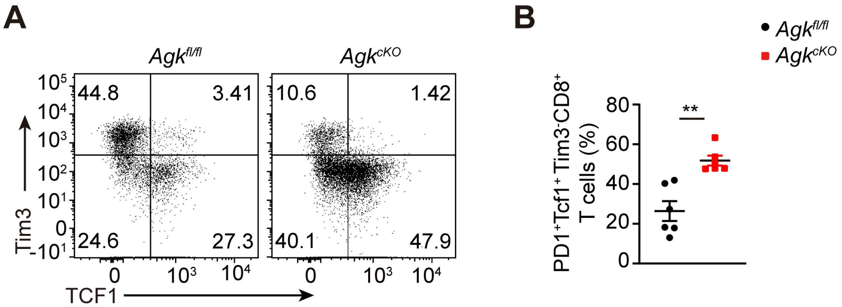
A
B
Agk^{fl/fl}
Agk^{cKO}
44.8
3.41
24.6
27.3
10.6
1.42
40.1
47.9
Tim3
TCF1
PD1+Tcf1+ Tim3-CD8+ T cells (%)
**
80
60
40
20
0

Figure S4

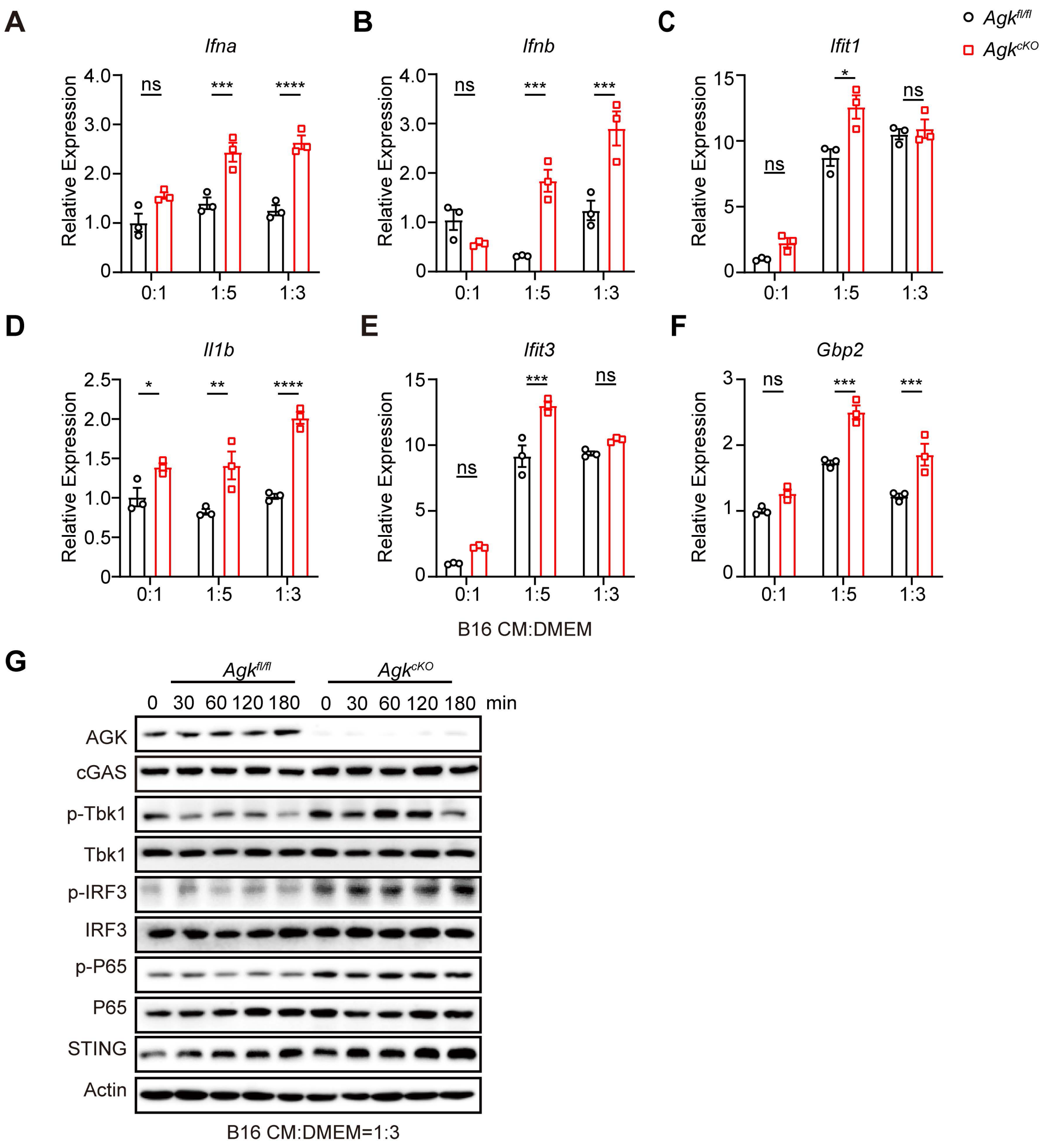
A
Ifna
B
Ifnb
C
Ifit1
Agk fl/fl
Agk cKO
Relative Expression
ns
***
****
*
0:1
1:5
1:3
D
Il1b
E
Ifit3
F
Gbp2
**
B16 CM:DMEM
G
0 30 60 120 180 0 30 60 120 180 min
AGK
cGAS
p-Tbk1
Tbk1
p-IRF3
IRF3
p-P65
P65
STING
Actin
B16 CM:DMEM=1:3

Figure S5

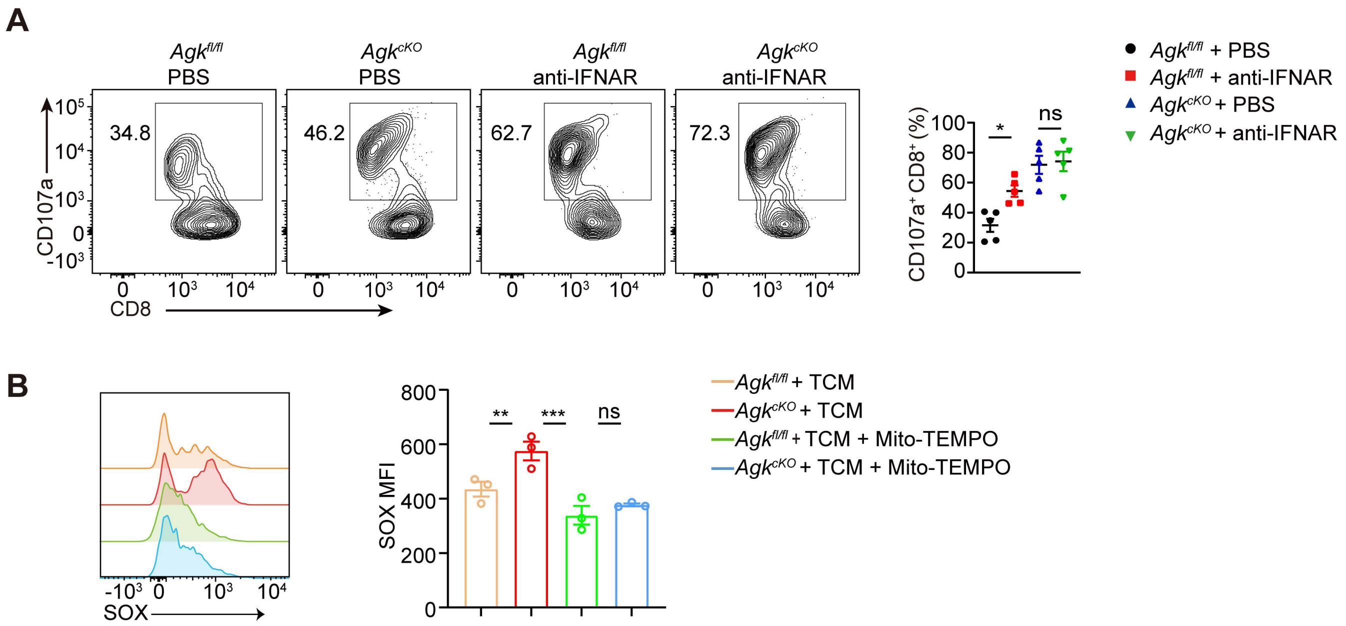
A
Agk fl/fl PBS
Agk cKO PBS
Agk fl/fl anti-IFNAR
Agk cKO anti-IFNAR
34.8
46.2
62.7
72.3
CD107a
CD8
CD107a+ CD8+ (%)
*
ns
Agk fl/fl + PBS
Agk fl/fl + anti-IFNAR
Agk cKO + PBS
Agk cKO + anti-IFNAR
B
SOX
SOX MFI
**
***
ns
Agk fl/fl + TCM
Agk cKO + TCM
Agk fl/fl + TCM + Mito-TEMPO
Agk cKO + TCM + Mito-TEMPO

Table S1. Oligonucleotide primers for qPCR

Target	Sequence
Mouse-Agk (F)	TTTGAGTCATACCCTCTTTGCC
Mouse-Agk (R)	TCAAGTGGAACTGTCTCTCCTT
Mouse-Ifna (F)	GCTTTCCTGATGGTTTTGGTG
Mouse-Ifna (R)	AGGCTTTCTTGTTCCTGAGG
Mouse-Ifnb (F)	CCAGCTCCAAGAAAGGACGA
Mouse-Ifnb (R)	TGGATGGCAAAGGCAGTGTA
Mouse-Ifit1 (F)	AGAGTCAAGGCAGGTTTCTG
Mouse-Ifit1 (R)	TGTGAAGTGACATCTCAGCTG
Mouse-Il1b (F)	GCAACTGTTCCTGAACTCAACT
Mouse-Il1b (R)	ATCTTTTGGGGTCCGTCAACT
Mouse-Ifit3 (F)	CTGAAGGGGAGCGATTGATT
Mouse-Ifit3 (R)	AACGGCACATGACCAAAGAGTAGA
Mouse-Gbp2 (F)	CTGCACTATGTGACGGAGCTA
Mouse-Gbp2 (R)	GAGTCCACACAAAGGTTGGAAA
Mouse-mtDNA Dloop1 (F)	AATCTACCATCCTCCGTGAAACC
Mouse-mtDNA Dloop1 (R)	TCAGTTTAGCTACCCCCAAGTTTAA
Mouse-mtDNA Dloop2 (F)	CCCTTCCCCATTTGGTCT
Mouse-mtDNA Dloop2 (R)	TGGTTTCACGGAGGATGG
Mouse-mtDNA 16S (F)	CACTGCCTGCCCAGTGA
Mouse-mtDNA 16S (R)	ATACCGCGGCCGTTAAA
Mouse-mtDNA ND4 (F)	AACGGATCCACAGCCGTA
Mouse-mtDNA ND4 (R)	AGTCCTCGGGCCATGATT
